# Investigation of epigenetic regulatory networks associated with autism spectrum disorder (ASD) by integrated global LINE-1 methylation and gene expression profiling analyses

**DOI:** 10.1371/journal.pone.0201071

**Published:** 2018-07-23

**Authors:** Chayanin Tangsuwansri, Thanit Saeliw, Surangrat Thongkorn, Weerasak Chonchaiya, Kanya Suphapeetiporn, Apiwat Mutirangura, Tewin Tencomnao, Valerie Wailin Hu, Tewarit Sarachana

**Affiliations:** 1 M.Sc. Program in Clinical Biochemistry and Molecular Medicine, Department of Clinical Chemistry, Faculty of Allied Health Sciences, Chulalongkorn University, Bangkok, Thailand; 2 Division of Growth and Development and Maximizing Thai Children’s Developmental Potential Research Unit, Department of Pediatrics, Faculty of Medicine, Chulalongkorn University and King Chulalongkorn Memorial Hospital, the Thai Red Cross Society, Bangkok, Thailand; 3 Center of Excellence for Medical Genetics, Department of Pediatrics, Faculty of Medicine, Chulalongkorn University, Bangkok, Thailand; 4 Excellence Center for Medical Genetics, King Chulalongkorn Memorial Hospital, the Thai Red Cross Society, Bangkok, Thailand; 5 Center of Excellence in Molecular Genetics of Cancer and Human Diseases, Department of Anatomy, Faculty of Medicine, Chulalongkorn University, Bangkok, Thailand; 6 Age-related Inflammation and Degeneration Research Unit, Department of Clinical Chemistry, Faculty of Allied Health Sciences, Chulalongkorn University, Bangkok, Thailand; 7 Department of Biochemistry and Molecular Medicine, The George Washington University School of Medicine and Health Sciences, Washington, DC, United States of America; Chiba Daigaku, JAPAN

## Abstract

**Background:**

The exact cause and mechanisms underlying the pathobiology of autism spectrum disorder (ASD) remain unclear. Dysregulation of long interspersed element-1 (LINE-1) has been reported in the brains of ASD-like mutant mice and ASD brain tissues. However, the role and methylation of LINE-1 in individuals with ASD remain unclear. In this study, we aimed to investigate whether LINE-1 insertion is associated with differentially expressed genes (DEGs) and to assess LINE-1 methylation in ASD.

**Methods:**

To identify DEGs associated with LINE-1 in ASD, we reanalyzed previously published transcriptome profiles and overlapped them with the list of LINE-1-containing genes from the TranspoGene database. An Ingenuity Pathway Analysis (IPA) of DEGs associated with LINE-1 insertion was conducted. DNA methylation of LINE-1 was assessed via combined bisulfite restriction analysis (COBRA) of lymphoblastoid cell lines from ASD individuals and unaffected individuals, and the methylation levels were correlated with the expression levels of LINE-1 and two LINE-1-inserted DEGs, *C1orf27* and *ARMC8*.

**Results:**

We found that LINE-1 insertion was significantly associated with DEGs in ASD. The IPA showed that LINE-1-inserted DEGs were associated with ASD-related mechanisms, including sex hormone receptor signaling and axon guidance signaling. Moreover, we observed that the LINE-1 methylation level was significantly reduced in lymphoblastoid cell lines from ASD individuals with severe language impairment and was inversely correlated with the transcript level. The methylation level of LINE-1 was also correlated with the expression of the LINE-1-inserted DEG *C1orf27* but not *ARMC8*.

**Conclusions:**

In ASD individuals with severe language impairment, LINE-1 methylation was reduced and correlated with the expression levels of LINE-1 and the LINE-1-inserted DEG *C1orf27*. Our findings highlight the association of LINE-1 with DEGs in ASD blood samples and warrant further investigation. The molecular mechanisms of LINE-1 and the effects of its methylation in ASD pathobiology deserve further study.

## Introduction

Autism spectrum disorder (ASD) is a group of neurodevelopmental disorders that are characterized by behavioral impairments, including deficits in social interaction and communication, and by restricted interests and repetitive behaviors. The Centers for Disease Control and Prevention (CDC) estimated that the prevalence of ASD is approximately 1 in 68 children in the United States and is likely to increase in the future [1]. Accumulating evidence indicates that ASD is caused by multiple genetic risk factors [2, 3], but abnormalities in the coding region of the genome are estimated to account for only 10–20% of all ASD individuals [4]. Other mechanisms, including epigenetic mechanisms, transcriptome profiles, and environmental factors, are therefore thought to contribute to the etiology and susceptibility to ASD.

Epigenetic mechanisms can induce changes in gene expression patterns while the genomic DNA sequence remains unaltered. These changes can be inherited and may be modulated by environmental factors, including diet, chemical exposures, and lifestyle [5]. DNA methylation is a common and important mechanism of epigenetic modification in mammalian genomes that can regulate gene expression. DNA methylation is the process of moving the methyl group from S-adenosyl-methionine (SAM) to cytosine by DNA methyltransferases (DNMT), usually at the fifth position of the cytosine residue in CpG dinucleotides, which are commonly found in promoter regions. Several studies have investigated DNA methylation profiles in ASD using tissues from various sources, including lymphoblastoid cell lines (LCLs) [6], peripheral blood [7], and post-mortem brain tissues [8, 9]. Ladd et al. (2014) and Nardone et al. (2014) identified differentially methylated regions and CpG sites in multiple brain regions of ASD and found that several differentially methylated genes were associated with ASD-related biological pathways [8, 9]. In addition, Nguyen et al. (2010) conducted a large-scale methylation profiling analysis of LCLs derived from monozygotic twins as well as sibling pairs discordant for a diagnosis of autism using CpG island microarrays [6]. Interestingly, they found that several genes, including *RORA* and *BCL2*, which are involved in biological processes associated with ASD, were differentially methylated in LCLs derived from ASD subjects. Moreover, they also found that RORA and BCL2 proteins were decreased in brain tissues of ASD individuals. These findings suggested that molecular changes observed in the peripheral blood or blood-derived cells may reflect some pathobiological conditions in the brain, further supporting the use of peripheral tissues as a surrogate to identify molecular marker candidates for ASD. However, these studies have focused mainly on the CpG islands of protein-coding regions, while DNA methylation in non-coding regions of the genome, which are thought to be involved in gene regulatory processes, has yet to be investigated.

Long interspersed element-1 (LINE-1) is a transposable element belonging to a group of non-long terminal repeat (non-LTR) retrotransposons. LINE-1 is an autonomous mobile element that comprises approximately 17% of the human genome where it remains active [10]. The full intact LINE-1 sequence is approximately 6,000 bp long and contains a 5' untranslated region (UTR) with an internal promoter, two non-overlapping open reading frames (ORF1 and ORF2), and a 3' UTR with a poly (A) tail [see review in [11]]. More than 500,000 copies of LINE-1 are present in the human genome [12] and among these, more than 10,000 LINE-1 copies are longer than 4.5 kb. These LINE-1 elements consist of a 5′ UTR, two open reading frames, and a 3′ UTR containing a poly (A) signal [13]. Retrotransposition of LINE-1 into a new genomic location may lead to duplication, deletion, or insertion of its nucleotide sequence at the target site, causing genomic instability and alteration of gene expression [14]. Evidence indicates that LINE-1 is involved in several processes, including double-strand break DNA repair [15], exon shuffling [16], gene silencing, and transgenesis processes [17]. Although regulation of LINE-1 transcription and retrotransposition remains unclear, accumulating evidence suggests that epigenetic mechanisms, including DNA methylation and histone modifications, are involved in the retrotransposition of LINE-1 and may impact the expression of target genes [18]. Aporntewan et al. (2011) found that hypomethylation of LINE-1 could repress the transcription of several genes related to cancer [19]. Moreover, altered DNA methylation levels and patterns of CpG residues in LINE-1 promoter regions have been reported in many diseases [20–23].

Accumulating evidence indicates that LINE-1 plays an important role in neurological functions [24]. Recent studies have demonstrated that the promoter of LINE-1 is transcriptionally activated during neuronal differentiation through the canonical WNT pathway, which is associated with the transition of neuronal stem cells, neurogenesis, and survival of neuronal progenitors [25–27]. LINE-1 contains CpG residues in the promoter region that can be regulated by Methyl-CpG binding Protein 2 (MeCP2), mutations of which are found in Rett syndrome, a severe developmental disorder with autistic phenotypes [28]. MeCP2 binds to methylated CpG residues and represses LINE-1 transcription in neural stem cells, and the transcription and retrotransposition of LINE-1 are increased in the absence of MeCP2 [28], suggesting that LINE-1 methylation plays an important role in the regulation of LINE-1 expression. Moreover, neural progenitor cells derived from iPSCs of individuals with Rett syndrome who carry MeCP2 mutations exhibit increased susceptibility to LINE-1 retrotransposition [28]. In addition to Rett syndrome, notably, ASD individuals who carry 15q11-q13 duplication have been reported to present lower levels of LINE-1 methylation in brain tissues [29]. Recently, Shpyleva et al. (2017) reported that LINE-1 is overexpressed and the binding of MeCP2 to LINE-1 sequences is significantly lower in the cerebellum of ASD individuals [30]. Through methylated DNA immunoprecipitation (MeDIP) analysis of cytosine-5 methylation in LINE-1, these authors also found that methylation of the LINE-1 5’UTR, ORF1, and ORF2 tended to be reduced in ASD, although this difference was not statistically significant [30], possibly due to heterogeneity within the ASD population.

To reduce the heterogeneity of ASD samples for transcriptomics analyses, Hu and Steinberg (2009) applied multiple clustering analyses to Autism Diagnostic Interview-Revised (ADI-R) scores for 123 items of nearly 1,954 individuals with ASD [31]. These multivariate clustering analyses showed that the ASD population could be divided into four phenotypic subgroups based on similarity of symptom severity across the 123 selected items. One of the clusters was characterized by severe language deficits, while the other three subgroups could be characterized by mild, intermediate, or savant phenotypes. To test the proof-of-principle that the phenotypic differences could be translated into differences in gene expression, ASD individuals from three subgroups were selected for transcriptome profiling analysis [32]. Interestingly, transcriptome profiling of LCLs derived from ASD individuals in each subgroup and from sex/age-matched unaffected individuals revealed differentially expressed genes unique to each phenotypic subgroup, as well as sets of overlapping differentially expressed genes among the subgroups [32]. More than 4,000 genes were reported to be significantly altered in the ASD subgroup with severe language deficits. The dysregulated genes included *RORA*, a gene encoding the nuclear receptor and transcription factor protein retinoic acid receptor (RAR)-related orphan receptor alpha, which exhibits the potential to transcriptionally regulate more than 2,500 ASD-associated genes and may play an important role in the sex bias of ASD [6, 32–36]. In addition to transcriptomic profiling, this same phenotype definition strategy has been employed in several genomic studies to identify novel ASD subtype-specific genetic variants and linkage regions as well as potential molecular markers for ASD [37–40]. These findings strongly suggest that using the phenotypic and/or clinical information of individuals to reduce the heterogeneity of ASD among subjects for ‘omics analyses may lead to a better understanding of ASD pathobiology and help identify biomarker candidates or potential therapeutic targets specific to the specific ASD sub-populations. Although LINE-1 methylation has been recently investigated in ASD as mentioned above, whether LINE-1 methylation is dysregulated in peripheral cells or blood-derived cells from all ASD individuals as opposed to specific phenotypic subgroups remains unclear. Moreover, whether LINE-1 insertion is associated with differentially expressed genes reported in ASD is still unknown.

**Fig 1:**
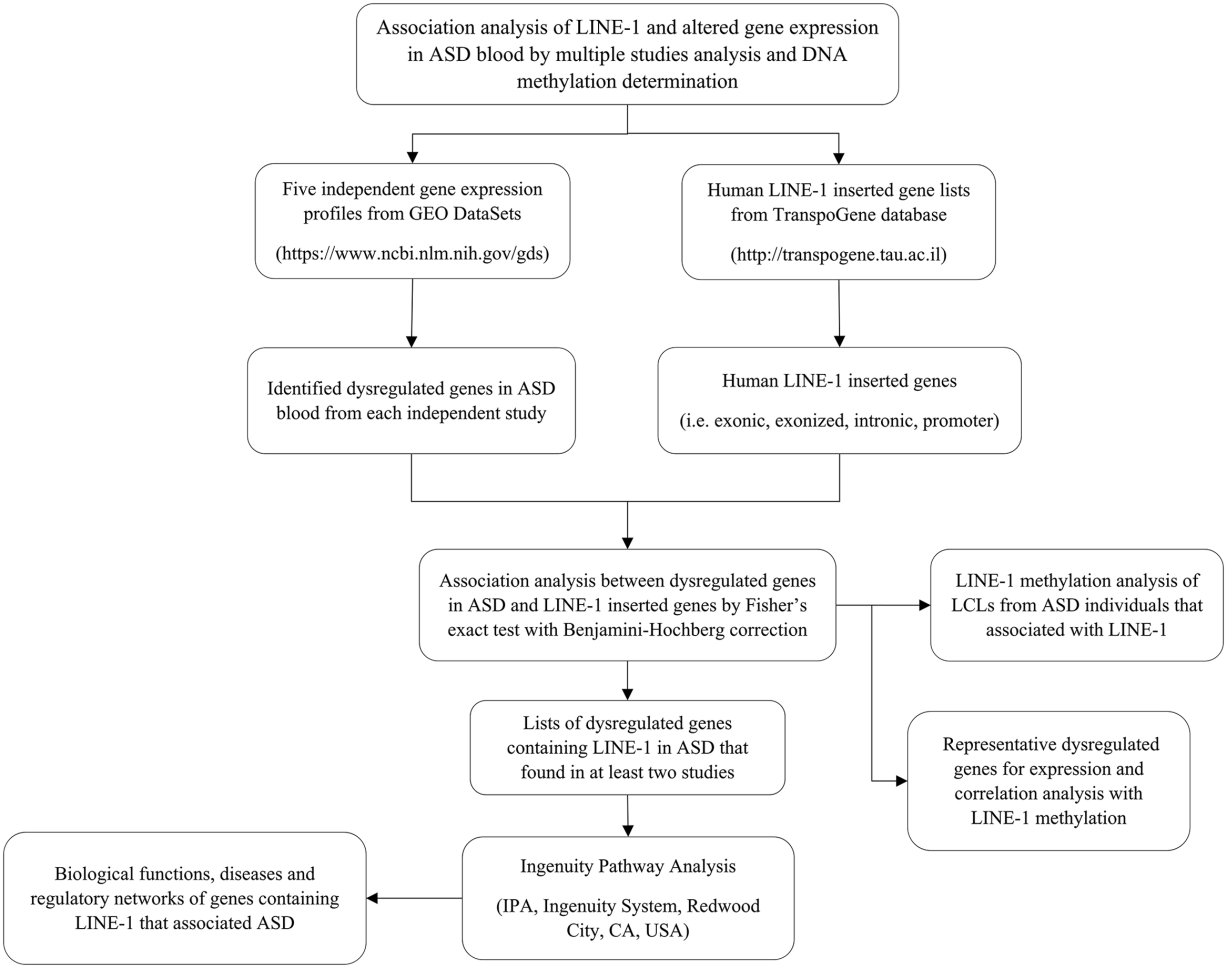
Schematic diagram of the experimental workflow of this study.

In this study, we sought to determine whether LINE-1 insertion is associated with differentially expressed genes reported in ASD. Moreover, we sought to measure LINE-1 methylation in lymphoblastoid cell lines from individuals with ASD compared to that in sex/age-matched unaffected individuals. To identify differentially expressed genes, we obtained and reanalyzed ASD transcriptome profiles from five previously published studies using data deposited in the NCBI GEO DataSets database. The list of differentially expressed genes from each study was then overlapped with the list of LINE-1-inserted genes from the TranspoGene database. Fisher’s exact test was applied to determine the probability of an association between LINE-1-inserted genes and the lists of differentially expressed genes. The list of differentially expressed genes with LINE-1 insertion that showed reproducibility in an independent study was then uploaded for Ingenuity Pathway Analysis to predict biological functions and pathways that may be impacted by LINE-1. We subsequently measured the methylation of LINE-1 in lymphoblasts from a combined group of ASD individuals, and also in a phenotypic subgroup of ASD individuals who exhibited severe language deficits as determined by the ADI-R clustering analysis approach, both of which were compared to the LINE-1 methylation state in lymphoblasts of sex/age-matched unaffected individuals. The level of LINE-1 methylation was also correlated with the expression of LINE-1 and the transcript levels of two differentially expressed genes with LINE-1 insertion. The experimental workflow of this study is illustrated in Fig 1.

## Materials and methods

### Collection of transcriptome profile data

To identify differentially expressed genes in ASD, the transcriptome profiles of ASD individuals and unaffected individuals were obtained from the NCBI Gene Expression Omnibus (GEO) DataSets database (http://www.ncbi.nlm.nih.gov/gds) [41, 42]. The transcriptomic studies selected for subsequent analyses had to meet the following criteria: i) the number of samples used in transcriptome profiling analysis corresponded to more than 40 individuals; ii) the analyzed samples were peripheral blood cells or cell lines derived from peripheral blood; and iii) transcriptome data and all supplementary information, including series matrix files and related platforms, must have been deposited in NCBI GEO DataSets and were available for reanalysis. Based on these criteria, five transcriptomic studies (i.e., GSE15402, GSE25507, GSE6575, GSE18123, and GSE42133) were selected. The details of each study are shown in Table 1.

**Table 1:** The details of the transcriptome profiles obtained from NCBI GEO DataSets database.

GEO accession number	Titles	Authors	Sample type	Sample size			References
				Controls	ASD	Total	
GSE 15402	Gene expression profiling differentiates autism case-controls and phenotypic variants of autism spectrum disorders	Hu et al. (2009)	Lymphoblastoid cell lines (LCLs)	29	87	116	[32]
GSE 25507	Autism and increased paternal age-related changes in global levels of gene expression regulation	Alter et al. (2011)	Peripheral blood lymphocytes	64	82	146	[43]
GSE 6575	Gene expression in blood of children with autism spectrum disorder	Gregg et al. (2008)	Peripheral blood	12	35	47	[44]
GSE 18123	Characteristics and predictive value of blood transcriptome signature in males with autism spectrum disorders	Kong et al. (2012)	Peripheral blood	115	170	285	[45]
GSE 42133	Cell cycle networks link gene expression dysregulation, mutation, and brain maldevelopment in autistic toddlers	Pramparo et al. (2015)	Peripheral blood lymphocytes	56	91	147	[46]

### Collection of LINE-1-inserted gene lists

The list of all LINE-1-inserted genes in Human Genome 18 (UCSC hg18, NCBI build 36.1) was downloaded from the TranspoGene database (http://transpogene.tau.ac.il) [47]. The list of LINE-1-inserted genes was also divided into different types of LINE-1 insertions (i.e., exonic, exonized, intronic, and promoter types). Multiple LINE-1 elements can insert into a single gene with different insertion types. For the LINE-1 inserted gene lists used in this study, we obtained all genes with at least one instance of one of the four insertion types.

The LINE-1-inserted genes used in our study were predicted by the TranspoGene database which may contain a full-length LINE-1 sequence and/or a conserved region of LINE-1. We obtained all genes that contained LINE-1 insertion in at least one element from the TranspoGene database, which covers transposable elements located inside human protein-coding genes. According to Asaf Levy et al. (2008), a LINE-1 sequence was retrieved from Human NCBI 36.1 (hg18) and overlapped with expressed sequence tags (ESTs) or cDNA alignments. Transposable elements with significant overlap were then identified and classified according to alignment location within the gene: proximal promoter, exonized (insertion within an intron that led to exon creation), exonic (insertion into an existing exon) or intronic transposable elements [47].

### Identification of differentially expressed genes and their association with LINE-1-inserted genes

To identify genes that are differentially expressed in ASD patient whole blood or blood-derived cells, transcriptome profiling data from each study obtained from NCBI GEO DataSets were analyzed separately using the Multiple Experiment Viewer (MeV) program (microarray software suit; http://mev.tm4.org) [48]. All transcriptome data were filtered with a 70% cut-off filter that removes transcripts for which data were missing in > 30% of samples. The available transcripts were then used for the identification of differentially expressed genes in ASD individuals compared with unaffected sex/age-matched controls using the two-tailed t-test with adjusted Bonferroni multiple test correction.

The list of differentially expressed genes from each study was then overlapped with the list of all human LINE-1-inserted genes, as well as the lists of human genes with different types of LINE-1 insertion. The overlapping genes were subsequently divided into 4 groups (i.e., dysregulated genes with LINE-1 insertion, dysregulated genes without LINE-1 insertion, non-dysregulated genes with LINE-1 insertion, and non-dysregulated genes without LINE-1 insertion) in a 2x2 table. Fisher’s exact tests were performed to determine whether LINE-1-inserted genes were significantly enriched in the lists of differentially expressed genes in ASD. Moreover, the differentially expressed genes were classified as downregulated or upregulated and used for association analysis with the human LINE-1-inserted gene lists. These procedures were repeated for each type of human LINE-1 insertion. A Fisher’s exact test P-value less than 0.05 after Benjamini Hochberg correction (FDR ≤ 0.05) was considered statistically significant. In addition, to further confirm that LINE-1 insertion was significantly associated with ASD-related DEGs, randomly chosen genes that were equal in number to that of DEGs from each study were used as a controls. Hypergeometric distribution analyses were then used to compare the statistical probability of LINE-1 insertion within the DEGs and randomly selected genes from each study. Moreover, the lists of differentially expressed genes with LINE-1 insertion from five transcriptome studies were then overlapped with one another using the Venn Diagram program (http://bioinformatics.psb.ugent.be/webtools/Venn). Differentially expressed genes with LINE-1 insertion reproducibly observed in at least two studies were selected for subsequent biological function and pathway analyses.

### Prediction of biological functions and networks associated with LINE-1-inserted differentially expressed genes in ASD

IPA software (Ingenuity System, Redwood City, CA, USA) was employed to identify biological functions, gene regulatory networks, diseases, and canonical pathways that are significantly associated with the dysregulated genes with LINE-1 insertion identified in at least two independent transcriptome studies. The significance of the association for each predicted biological function or canonical pathway was determined with Fisher’s exact test (P-value < 0.05).

### Phenotypic subgrouping of ASD

A total of 56 lymphoblastoid cell lines (LCLs) derived from peripheral mononuclear cells of ASD subjects (n = 36) and sex-/age-matched unaffected individuals (n = 20) were used for this study. These individuals were previously assigned to several phenotypic subgroups within ASD based on cluster analyses of ADI-R scores, as previously described in detail [31]. The exclusion criteria for this study included cognitive impairment (Raven’s scores < 70), the presence of known genetic abnormalities (i.e., fragile X, Rett syndrome, tuberous sclerosis, or chromosome 15q11-q13 duplication), premature birth (< 35 weeks gestation), and diagnosis of comorbid psychiatric disorders (i.e., bipolar disorder, obsessive compulsive disorder, or severe anxiety). Impairment in spoken language was also confirmed based on low standard scores (< 80) on the Peabody Picture Vocabulary Test. These exclusion criteria are expected to reduce the heterogeneity of subjects to study idiopathic ASD. The LCLs used in this study represented individuals from three phenotypic groups based on clustering analysis: severe language impairment (subgroup L); milder symptoms (subgroup M); and savant skills (subgroup S) based on multivariate cluster analyses of ADI-R scores. These LCLs have been previously used in a transcriptome profiling study by Hu et al. (2009) [32], which is one of the studies that we employed for the identification of differentially expressed genes in ASD (GEO accession number GSE15402). The demographic information of the individuals whose LCLs were used in this study is shown in S1 Table.

### Cell culture

All LCLs were obtained from the Autism Genetics Resource Exchange Repository (AGRE, Los Angeles, CA, USA) and cultured according to the protocol of the Rutgers University Cell and DNA Repository, which produces and maintains cells in the AGRE collection as previously described [32]. Briefly, all LCLs were cultured in RPMI 1640 medium supplemented with 15% fetal bovine serum and 1% penicillin/streptomycin under optimum conditions, at 37°C with 5% carbon dioxide. The cultured cell lines were split 1:2 every three to four days, and cells were harvested for analysis on day three after splitting, when the cultures were in the logarithmic growth phase. After harvesting, all cell lines were stored in RNAlater (Applied Biosystem) at -80°C to maintain the genetic material until further analysis. Genomic DNA and total RNA were isolated from the LCLs of ASD individuals and unaffected sex/age-matched controls as previously described [49, 50]. The concentrations of DNA and RNA were measured using a NanoDrop 1000 spectrophotometer (Thermo Scientific, USA).

### Combined bisulfite restriction analysis (COBRA) of LINE-1 methylation

The methylation level of LINE-1 was measured using COBRA as described in previous reports [21, 22, 51, 52]. Briefly, genomic DNA was treated with sodium bisulfite using the EZ DNA Methylation-Gold^™^ Kit (Zymo Research, Irving, CA, USA) according to the manufacturer’s protocol. The bisulfite-converted DNA was then employed as a template in PCR using HotStarTaq DNA polymerase (QIAGEN, USA), the forward primer (5'-CCGTAAGGGGTTAGGGAGTTTTT-3'), and the reverse primer (5'-RTAAAACCCTCCRAACCAAATATAAA-3'), which are specific to the regulatory region of LINE-1 (based on GenBank: M80343), at a denaturing temperature of 95°C, annealing temperature of 50°C, and extension temperature of 72°C. After amplification, the LINE-1 amplicons (160 bp) were digested at 65°C overnight in NEB buffer 3 with the TaqI and TasI restriction enzymes (Thermo Scientific, USA), which can specifically cut at methylated cytosines and unmethylated cytosines, respectively. The digested LINE-1 amplicons were separated in an 8% non-denaturing polyacrylamide gel. Band intensities were measured and analyzed as described previously [20, 49, 50].

Based on the DNA methylation patterns of the 2 CpG dinucleotides, the LINE-1 amplicons from COBRA produced 4 bands corresponding to hypermethylated loci (C^m^ C^m^), hypomethylated loci (C^u^ C^u^), and 2 partially methylated loci (C^m^ C^u^ and C^u^ C^m^) in which the sizes of each product were 160 bp, 98 bp, 80 bp and 62 bp, respectively. To determine the accurate percentage of CpG dinucleotides, we calculated the LINE-1 methylation level of each pattern as described in a previous study [20]. Briefly, the band intensity of each product was determined as follows: % intensity of the 160-bp product/160 = A, % intensity of the 98-bp product/98 = B, % intensity of the 80-bp product/80 = C, and % intensity of the 62-63-bp product/62 = D. The percentage of the overall methylation levels of two CpG sites within LINE-1 was then calculated using the following formula: % C^m^ = 100x(C+A)/(C+A+A+B+D), and the LINE-1 methylation levels of each pattern were estimated by following these principles: % C^m^ C^u^ = 100x(A)/([(C-D+B)/2]+A+D), % C^u^ C^m^ = 100x(D-B)/[(C-D+B)/2]+A+D, % C^u^ C^u^ = 100xB/([(C-D+B)/2]+A+D), and % C^m^ C^m^ = 100x[(C-D+B)/2]/([(C-D+B)/2]+D+A) according to previous studies [20].

### Quantitative reverse transcription-PCR analysis

Total RNA was treated with DNase prior to qPCR analysis using RQ1 RNase-Free DNase (Promega) according to the manufacturer’s protocol. DNase-treated RNA was then subjected to qRT-PCR analysis. Briefly, 1 μg of DNase-treated RNA was converted to cDNA using 0.5 μg of primer (oligo dT_18_) and AccuPower^®^ RT PreMix (Bioneer) according to the manufacturer’s recommended protocol. Real-time PCR analysis was performed with AccuPower^®^ 2X GreenStar^™^ qPCR MasterMix (Bioneer) using 1 μl of cDNA according to the manufacturer’s protocol. The primer sequences employed in this study were as follows: LINE-1 forward primer, 5'- CACTAGGGAGTGCCAGACA-3' and reverse primer, 5'- GACCGTCACCCCTTTCTT-3'; *C1orf27* forward primer, 5'-CAAGCTTCATTGGACAACACA-3' and reverse primer, 5'-AGATACCCGCAGCAAGGAC-3'; and *ARMC8* forward primer, 5'-CCTCATTGCCATGCTTGCTG-3' and reverse primer, 5'-CGGAGTTCGTGAGCATGTTTT-3'. The levels of LINE-1 and selected gene transcripts were normalized to the housekeeping gene *GAPDH* (forward primer, 5’-ATGTTCGTCATGGGTGTGAA-3’ and reverse primer, 5’-ACAGTCTTCTGGGTGGCAGT-3’). The qPCR conditions were set as follows: for LINE1 and *C1orf27*, pre-denaturation step at 95°C for 15 minutes, 40 cycles of denaturation at 95°C for 15 seconds, annealing at 60°C for 30 seconds, and extension at 72°C for 30 seconds; for *ARMC8*, pre-denaturation at 95°C for 15 minutes, 45 cycles of denaturation at 95°C for 15 seconds, annealing at 58°C for 15 seconds, and extension at 72°C for 30 seconds. A melting curve analysis was performed to confirm product formation. The expression levels of LINE-1 and the other genes were calculated using the 2^-ΔΔ^Ct method.

### Statistical analyses

The two-tailed t-test with adjusted Bonferroni correction was employed to determine differentially expressed genes in each transcriptomic analysis. Adjusted P-values less than 0.05 were considered statistically significant. The associations between the lists of dysregulated genes in ASD and the lists of human LINE-1-inserted genes were determined using Fisher’s exact test with Benjamini-Hochberg correction (FDR = 0.05). Adjusted P-values less than 0.05 were considered statistically significant. Pathway and function analyses were performed with IPA using Fisher’s exact test, and P-values less than 0.05 were considered significant. Two-tailed t-tests were used to analyze differences in LINE-1 methylation levels. Benjamini-Hochberg correction (FDR = 0.05) was applied to adjust P-values in multiple ASD phenotypic subgroup comparisons. Correlations between LINE-1 methylation levels and the expression levels of LINE-1, *C1orf27*, and *ARMC8* were assessed using Spearman’s correlation and P-values less than 0.05 were considered significant.

### Ethics statement

The use of lymphoblastoid cell lines in this study was reviewed by the GWU Office of Human Research and determined to be “Exempt” from full IRB review for human subjects research because all cell lines and phenotypic data used in this study were de-identified with respect to donor, and there were never any direct interactions with any humans whose cells were used in this study.

## Results

### Genes containing LINE-1 insertion are differentially expressed in whole blood and blood-derived cells of ASD individuals

To determine whether LINE-1 insertion is associated with dysregulated gene expression in peripheral blood and blood-derived cells from ASD individuals, we assessed the associations between the lists of LINE-1-inserted genes and differentially expressed genes in ASD. The transcriptome profiles of ASD individuals and unaffected sex/age-matched controls were obtained from five independent ASD studies (Table 1) and reanalyzed. The differentially expressed genes in ASD detected in each study were identified. The list of human genes with LINE-1 insertions was obtained from the TranspoGene database and then overlapped with the list of dysregulated genes in ASD from each study. Interestingly, an association analysis using Fisher’s exact test revealed that genes containing LINE-1, regardless of the insertion type (i.e., “all insertion”), were overrepresented among dysregulated genes in ASD whole blood or blood cell samples in four of the five transcriptomic studies (P-value < 0.05; Table 2). The lists of differentially expressed genes with LINE-1 insertion consisted of 1,013, 836, 530, and 312 genes from the GSE18123, GSE25507, GSE42133, and GSE6575 studies, respectively. Next, we categorized the dysregulated genes into up- and downregulated genes and subsequently identified the overlap with LINE-1-inserted genes to determine whether the overrepresentation of LINE-1-inserted genes was biased towards up- or downregulated genes in ASD. The results showed that LINE-1-inserted genes were significantly enriched in both upregulated and downregulated genes (Table 2). Moreover, to predict the types of LINE-1 insertion that are associated with differentially expressed genes in ASD, the lists of genes with specific LINE-1 insertion types (i.e., intronic, exonized, exonic, and promoter types) were produced and then overlapped with the dysregulated genes identified from each transcriptomic study. Interestingly, only LINE-1 intronic insertion was found to be strongly associated with dysregulated genes in ASD (Table 2). To further determine whether LINE-1-inserted genes were significantly over-represented within the lists of DEGs from each study, we also performed hypergeometric distribution analyses using randomly chosen genes from each study that were equal in number to the up- and down-regulated DEGs from each study. P-values were then calculated and adjusted using the Benjamini-Hochberg correction method for multiple tests (FDR < 0.05). Interestingly, almost all DEG lists which showed enrichment in known LINE-1-inserted genes had a P-value much lower than that of the list of randomly chosen genes, suggesting that there are significantly more LINE-1 insertions in ASD-associated DEGs than in randomly chosen genes. The results of hypergeometric distribution analysis are shown in S2 and S3 Tables.

**Table 2:** Association analyses between the differentially expressed genes in ASD and the human LINE-1-inserted gene lists.

Insertion type	Comparison	GEO datasets	All dysregulated genes		Upregulated genes		Downregulated genes	
			P-value	Genes (n)	P-value	Genes (n)	P-value	Genes (n)
All insertion	ASD vs. Control	GSE15402	0.503	134	0.550	62	0.821	73
		GSE18123	**<0.0001**	1,013	**<0.0001**	849	0.098	172
		GSE25507	**0.001**	530	0.352	267	**<0.0001**	265
		GSE42133	**<0.0001**	836	**0.009**	493	**0.004**	346
		GSE6575	**<0.0001**	312	**<0.0001**	161	0.503	153
Intronic	ASD vs. Control	GSE15402	0.550	133	0.550	61	0.908	73
		GSE18123	**<0.0001**	1,003	**<0.0001**	839	0.196	172
		GSE25507	**0.001**	528	0.503	266	**<0.0001**	264
		GSE42133	**<0.0001**	825	**0.010**	484	**0.002**	344
		GSE6575	**<0.0001**	311	**<0.0001**	160	0.352	153
Exonized	ASD vs. Control	GSE15402	0.503	0	1.000	0	0.821	0
		GSE18123	0.352	15	0.503	11	0.556	4
		GSE25507	0.652	8	0.444	1	**0.039**	7
		GSE42133	0.719	12	0.352	10	0.679	2
		GSE6575	1.000	3	0.652	0	0.678	3
Exonic	ASD vs. Control	GSE15402	1.000	6	0.751	4	0.908	2
		GSE18123	0.503	39	**0.033**	38	**0.015**	1
		GSE25507	0.292	12	0.055	4	0.983	8
		GSE42133	0.503	37	0.904	19	0.352	18
		GSE6575	0.993	11	1.000	4	0.856	7
Promoter	ASD vs. Control	GSE15402	1.000	1	1.000	0	0.821	1
		GSE18123	0.503	8	0.719	7	0.908	2
		GSE25507	0.679	5	0.821	3	0.908	2
		GSE42133	0.908	14	1.000	7	0.719	7
		GSE6575	0.550	6	0.339	4	1.000	2

The list of dysregulated genes in ASD from each transcriptomic study was overlapped with the list of LINE-1-inserted genes. The significance of the association between gene sets was determined using Fisher’s exact test with Benjamini-Hochberg correction (FDR = 0.05). Adjusted P-values less than 0.05 were considered significant. The number of dysregulated genes with LINE-1 insertion and adjusted P-values are shown.

Previous studies have demonstrated that phenotypic subgrouping of individuals with idiopathic ASD allowed for identification of genes associated with specific ASD sub-populations [31, 37–40]. To determine whether LINE-1-inserted genes are overrepresented in specific phenotypic subgroups of ASD, we reanalyzed the transcriptome dataset GSE15402 by dividing ASD individuals into subgroups based on multivariate cluster analyses of ADI-R scores of ASD individuals as conducted by Hu and Steinberg [31]. This method of subphenotyping of individuals with ASD revealed that the ASD population could be represented by four phenotypic subgroups: i) the L subgroup was characterized by severe language deficits in addition to an overall higher severity in most ADI-R items; ii) the M subgroup, despite the ASD diagnosis, exhibited milder symptoms across various behavioral impairments; iii) the S subgroup possessed a noticeably higher frequency of savant skills; and iv) the Intermediate (I) group exhibited intermediate severity across all domains [31]. Here, we conducted an association analysis of the DEGs identified in transcriptomic analyses of three of the subtypes (L, M, and S) and LINE-1-inserted genes (Table 3). Interestingly, LINE-1-inserted genes were found to be over-represented only in down-regulated DEGs in the L subgroup with severe language deficits.

**Table 3:** Association analyses between the differentially expressed genes in ASD phenotypic subgroups and the human LINE-1-inserted gene lists.

Insertion type	Comparison	Differential gene expression		Upregulated gene expression		Downregulated gene expression	
		P-value	Genes (n)	P-value	Genes (n)	P-value	Genes (n)
All insertion	Subgroup L vs. control	0.492	686	0.738	305	**0.027**	387
	Subgroup M vs. control	0.578	265	0.613	171	0.738	96
	Subgroup S vs. control	0.738	115	0.730	63	1.000	52
Intronic	Subgroup L vs. control	0.499	674	0.738	300	**0.027**	380
	Subgroup M vs. control	0.578	261	0.613	169	0.738	94
	Subgroup S vs. control	0.738	113	0.613	63	0.903	50
Exonized	Subgroup L vs. control	0.738	12	0.492	9	0.738	3
	Subgroup M vs. control	0.940	2	0.738	0	0.738	2
	Subgroup S vs. control	0.877	2	0.760	1	0.752	1
Exonic	Subgroup L vs. control	0.644	35	0.738	17	0.738	18
	Subgroup M vs. control	1.000	10	1.000	6	0.941	4
	Subgroup S vs. control	0.613	8	1.000	2	0.392	6
Promoter	Subgroup L vs. control	0.738	11	0.820	5	0.738	6
	Subgroup M vs. control	1.000	3	0.905	1	0.738	2
	Subgroup S vs. control	1.000	1	1.000	0	0.738	1

The list of differentially expressed genes in ASD from the GSE15402 transcriptomic study was obtained and reanalyzed separately based on each phenotypic subgroup. The significance of the association was determined using Fisher’s exact test with Benjamini-Hochberg correction (FDR = 0.05). Adjusted P-values less than 0.05 were considered significant. The number of dysregulated genes with LINE-1 insertion and adjusted P-values are shown.

**Fig 2:**
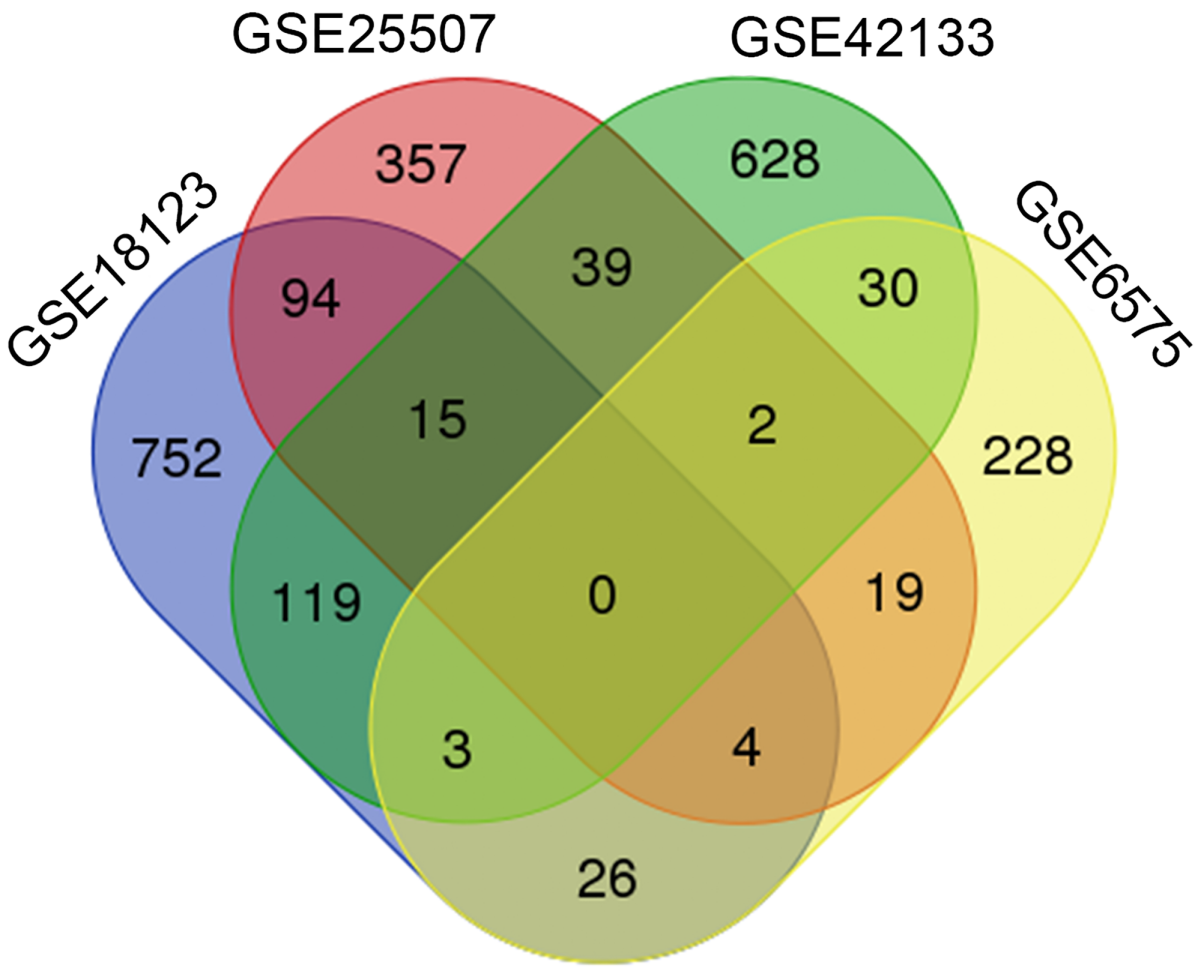
Venn diagram of LINE-1-inserted genes that are differentially expressed in ASD. The lists of significantly differentially expressed genes with LINE-1 insertions from each transcriptomic study were overlapped with one another. A total of 351 genes were found to be overlapping in at least two studies.

Moreover, to determine whether the dysregulated genes containing LINE-1 insertion are shared common among independent ASD studies, overlap analysis of the dysregulated genes containing LINE-1 insertion from each transcriptomic study was performed (Fig 2). We found that as many as 351 LINE-1-inserted genes were differentially expressed in ASD in at least two independent transcriptomic studies. Genes in each section of the Venn diagram are listed in S4 Table.

### Differentially expressed LINE-1-inserted genes in ASD are associated with neurological functions and disorders

**Fig 3:**
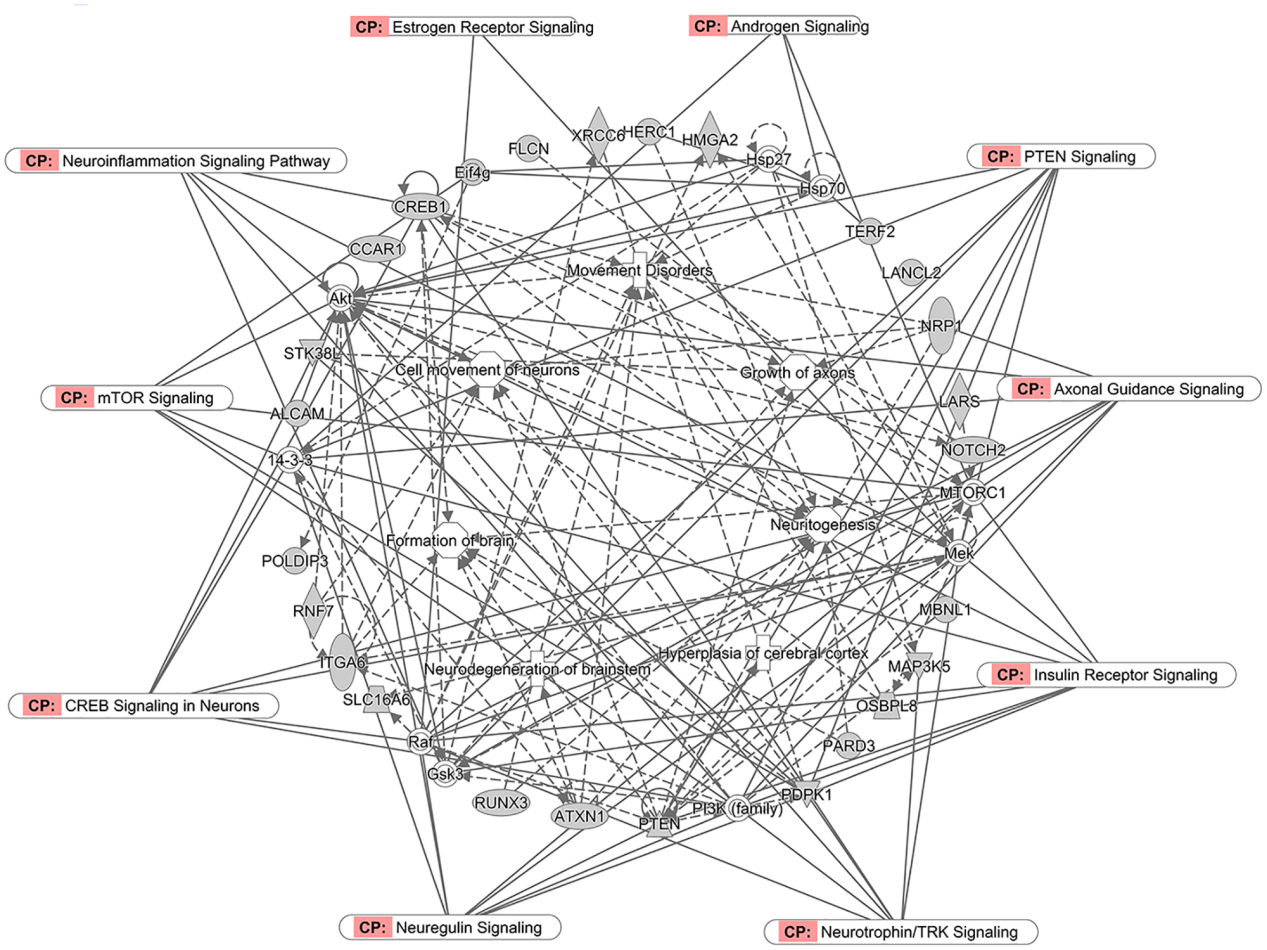
Predicted gene regulatory network of dysregulated genes with LINE-1 insertion predicted via IPA. The differentially expressed genes with LINE-1 insertion that were identified in multiple studies were used to create gene regulatory networks. The genes present in our dataset (shown in gray) were predicted to interact with one another and were associated with neurological functions known to be associated with ASD.

IPA software was employed to predict biological functions, disorders, networks, and pathways significantly associated with LINE-1-inserted genes that are dysregulated in ASD. IPA biological function and disease analyses revealed that differentially expressed genes with LINE-1 insertion (351 genes) were significantly associated with neurological functions and disorders (P-value < 0.05, Table 4). As many as 25 genes were significantly associated with autism or intellectual disability (P-value = 7.05E-06). Interestingly, neurodevelopmental disorders comorbid with ASD, including mental retardation, cognitive impairment, and neuromuscular disease, were also found to be significantly associated with this gene set (P-value < 0.05). Moreover, an IPA canonical pathway analysis of the differentially expressed genes containing LINE-1 insertion revealed a significant association with several biological functions, including ERK/MAPK signaling [53], axonal guidance signaling [54, 55], neurotrophin/TRK signaling [56], synaptic long-term potentiation, CREB signaling in neurons [see review in [57]], estrogen receptor signaling [33, 58, 59], androgen signaling [33], PTEN signaling [60], mTOR signaling [61], circadian rhythm signaling [[62] and see review in [63]], and neuroinflammation signaling [64] (P-value < 0.05, Table 5), all of which have been implicated in ASD. The gene regulatory network analysis also revealed complex interactions between the genes and neurological functions including neuritogenesis, hyperplasia of the cerebral cortex, neurodegeneration, and movement disorder (Fig 3).

**Table 4:** Developmental and neurological diseases significantly associated with dysregulated genes with LINE-1 insertion predicted via IPA.

Diseases	P-value	Genes (n)	Gene symbol
Autism or intellectual disability	7.05E-06	25	ADNP, ADRA1A, ARID1A, CAMTA1, CDC42, CREBBP, CSNK2A1, DYRK1A, ELOVL4, EP300, GNB1, GNB5, GRIK2, HERC1, NRP2, PTEN, RNF125, SMARCA2, SON, ST3GAL3, SYNE1, TRIO, WASHC4, YY1, ZBTB16
Familial mental retardation	4.61E-06	22	ADNP, ADRA1A, ARID1A, CAMTA1, CDC42, CREBBP, CSNK2A1, DYRK1A, ELOVL4, EP300, GNB1, GNB5, GRIK2, HERC1, RNF125, SMARCA2, SON, ST3GAL3, TRIO, WASHC4, YY1, ZBTB16
Mental retardation	5.75E-06	24	ADNP, ADRA1A, ARID1A, CAMTA1, CDC42, CREBBP, CSNK2A1, DYRK1A, ELOVL4, EP300, GNB1, GNB5, GRIK2, HERC1, NRP2, RNF125, SMARCA2, SON, ST3GAL3, SYNE1, TRIO, WASHC4, YY1, ZBTB16
Neurodevelopmental disorder	3.96E-03	7	ADRA1A, AHNAK, ASXL2, CREBBP, ESR1, HECW2, NF1
Cognitive impairment	5.01E-06	27	ADNP, ADRA1A, ARID1A, BCL2, CAMTA1, CDC42, CREBBP, CSNK2A1, DYRK1A, ELOVL4, EP300, ESR1, GNB1, GNB5, GRIK2, HECW2, HERC1, NRP2, RNF125, SMARCA2, SON, ST3GAL3, SYNE1, TRIO, WASHC4, YY1, ZBTB16
Early-onset neurological disorder	4.27E-03	9	ADRA1A, GNAO1, GRIK2, HGF, ITGA6, MYH9, OTOF, SPTAN1, ST3GAL3
Brain lesion	1.50E-03	35	ADRA1A, ANXA7, ARID1A, CBL, CD44, CREBBP, CTBP2, DICER1, EP300, EPHA1, EPHA8, ESR1, HDAC3, HERC1, HGF, LRIG2, LYST, MAML3, NF1, NRP2, PABPC1, PDPK1, PRKCH, PTEN, PTPN11, RECK, SAP130, SON, SYNE1, TERF2, TOP1, TRIM33, TRIP11, ZCCHC6, ZNF91
Neuromuscular disease	1.02E-03	35	ADAMTSL1, ADRA1A, ALCAM, ATP2A2, ATXN1, BCL2, CANX, CD44, CSN3, DCK, ESR1, GNAO1, GNB5, GPR107, GRIK2, ICA1, IFNAR2, MBP, MBTPS1, NOTCH2, OSBPL8, PPP3CB, PTPN3, PTPRC, PTPRE, RUNX3, SPAG16, SSX2IP, ST8SIA4, TLR2, TRIO, VDR, WNK1, XRCC6, ZBTB16

The differentially expressed genes with LINE-1 insertion that were identified in multiple studies were employed for IPA analysis. P-values calculated by Fisher’s exact test and the number of genes for each function are shown.

**Table 5:** Canonical pathways significantly associated with dysregulated genes with LINE-1 insertion predicted by the Ingenuity Pathway Analysis (IPA).

Canonical Pathways	P-value	Gene Symbol
ERK/MAPK signaling	1.45E-05	PLA2G2D, PAK2, CREB1, EP300, ELF2, PRKAG2, PPP2R2C, PTPN11, PPP1CB, RAP1B, MAPK1, CREBBP, ETS1, ESR1
HGF signaling	3.80E-05	CDC42, MAP3K5, PTPN11, RAP1B, MAPK1, ELF2, PRKCH, MAP3K4, ETS1, HGF
Axonal Guidance signaling	5.50E-05	SEMA4D, CDC42, PAK2, ACTR2, GNAO1, NRP2, PAPPA, PLXNC1, GNB1, PPP3CB, PRKAG2, EPHA1, GNB5, PTPN11, NRP1, SEMA7A, RAP1B, MAPK1, PRKCH, EPHA8, WNT9A
Neurotrophin/TRK signaling	6.76E-05	CDC42, MAP3K5, PDPK1, CREB1, PTPN11, EP300, MAPK1, CREBBP
Huntington’s Disease signaling	1.51E-04	CREB1, EP300, GNB1, HDAC3, SP1, TAF4, RCOR3, PDPK1, GNB5, PTPN11, MAPK1, CREBBP, PRKCH, CASP4
Synaptic long-term potentiation	2.88E-04	CREB1, EP300, PPP1CB, RAP1B, PPP3CB, MAPK1, CREBBP, PRKAG2, PRKCH
CREB signaling in neurons	4.07E-04	CREB1, GNB5, GNAO1, PTPN11, CACNG6, GRIK2, EP300, GNB1, MAPK1, CREBBP, PRKAG2, PRKCH
ILK signaling	7.24E-04	CDC42, PTEN, PDPK1, CREB1, PTPN11, EP300, MAPK1, CREBBP, ACTN1, PPP2R2C, MYH9
Androgen signaling	7.59E-04	GNB5, GNAO1, CACNG6, EP300, GNB1, MAPK1, CREBBP, PRKAG2, PRKCH
PTEN signaling	1.32E-03	CDC42, PTEN, PDPK1, INPP5F, BCL2, MAPK1, CBL, CSNK2A1
PI3K/AKT signaling	6.61E-03	MAP3K5, PTEN, PDPK1, INPP5F, BCL2, MAPK1, PPP2R2C
Estrogen receptor signaling	8.13E-03	TAF4, CTBP2, EP300, MAPK1, CREBBP, HDAC3, ESR1
Protein ubiquitination pathway	2.04E-02	UBR2, UBE2B, USP25, UBE4B, UBR1, USP31, CBL, USP33, USP6, USP47
Circadian rhythm signaling	2.24E-02	CREB1, EP300, CREBBP
mTOR signaling	2.51E-02	PDPK1, PTPN11, EIF4B, EIF4G3, MAPK1, PRKAG2, PRKCH, PPP2R2C
Neuroinflammation signaling pathway	4.47E-02	PLA2G2D, TLR8, CREB1, PTPN11, BCL2, EP300, PPP3CB, MAPK1, CREBBP, TLR2

The differentially expressed genes with LINE-1 insertion that were identified in multiple studies were employed for IPA analysis. P-values calculated by Fisher’s exact test and the number of genes for each canonical pathway are shown.

### Hypomethylation of LINE-1 in ASD with severe language deficits

**Fig 4:**
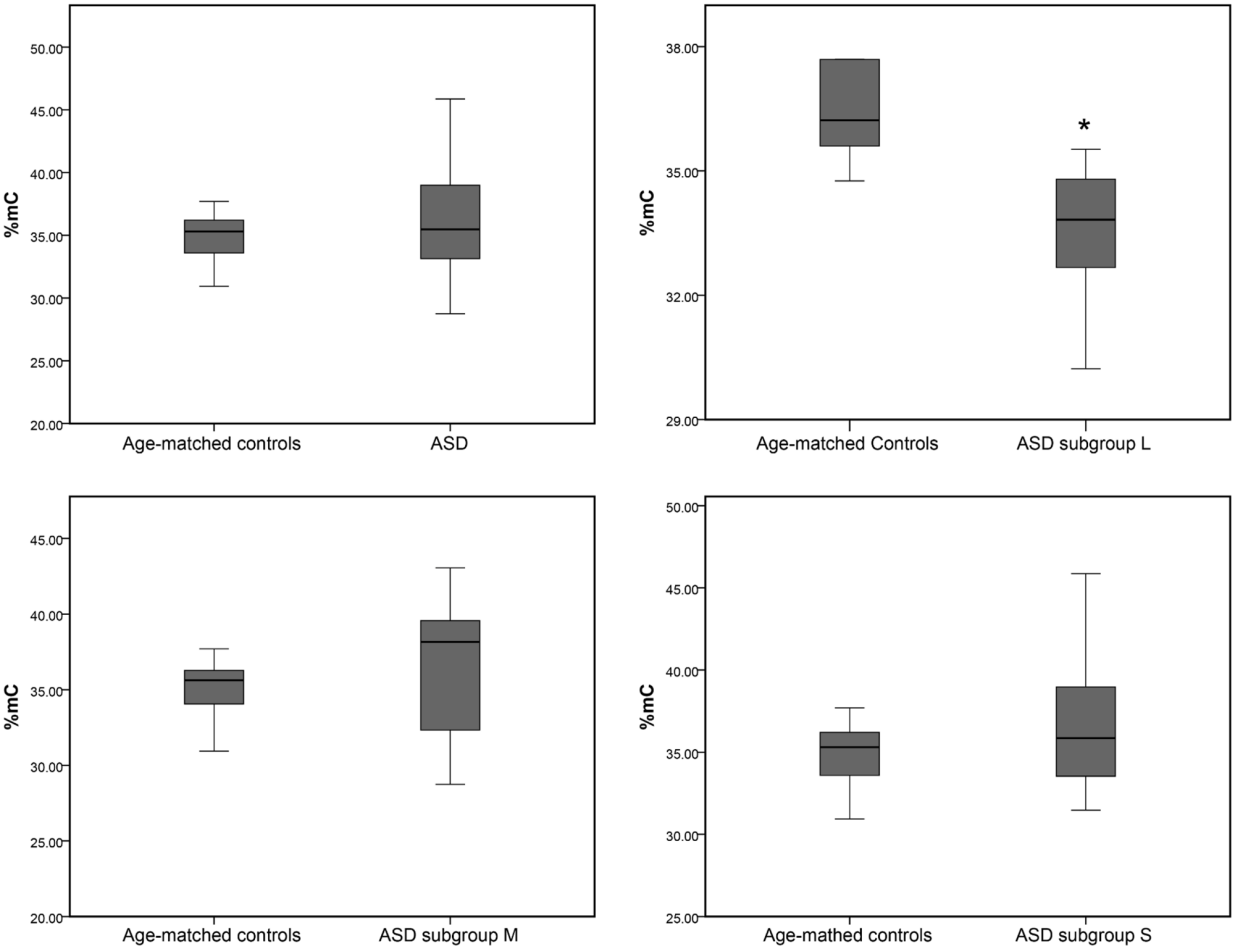
Box plot of the overall LINE-1 methylation level (%C^m^) between the ASD and sex/age-matched control groups. P-values were calculated via the two-tailed t-test with Benjamini-Hochberg correction for multiple subgroup comparisons.

The results of our association analyses and biological function/network analyses strongly suggested that LINE-1 insertion may be associated with ASD, providing a rationale for further investigation of LINE-1. Recent studies have shown that LINE-1 methylation is dysregulated in several diseases and may serve as a promising biomarker candidate [21, 49]; however, methylation of LINE-1 in ASD peripheral cells has not been investigated. In the current study, COBRA assays were performed to measure LINE-1 methylation levels and patterns in LCLs derived from ASD and unaffected sex-/age-matched individuals. The percentage of overall LINE-1 methylation (%C^m^) was not significantly altered in ASD individuals compared with that in the controls (Fig 4). Because the association analysis revealed that LINE-1 insertion is significantly associated with decreased gene expression only in LCLs from ASD individuals with severe language deficits, we further classified ASD into phenotypic subgroups for methylation analyses. COBRA analysis showed that the percentage of overall LINE-1 methylation (%C^m^) was significantly decreased (33.48% ± 1.92%, P-value = 0.039) in ASD with severe language deficits (Subgroup L), but not in the other two subgroups, compared to that in age/sex-matched unaffected controls (36.37% ± 1.16%, Fig 4).

### Global LINE-1 methylation levels are correlated with the expression of LINE-1 and LINE-1-inserted genes in ASD with severe language deficits

**Fig 5:**
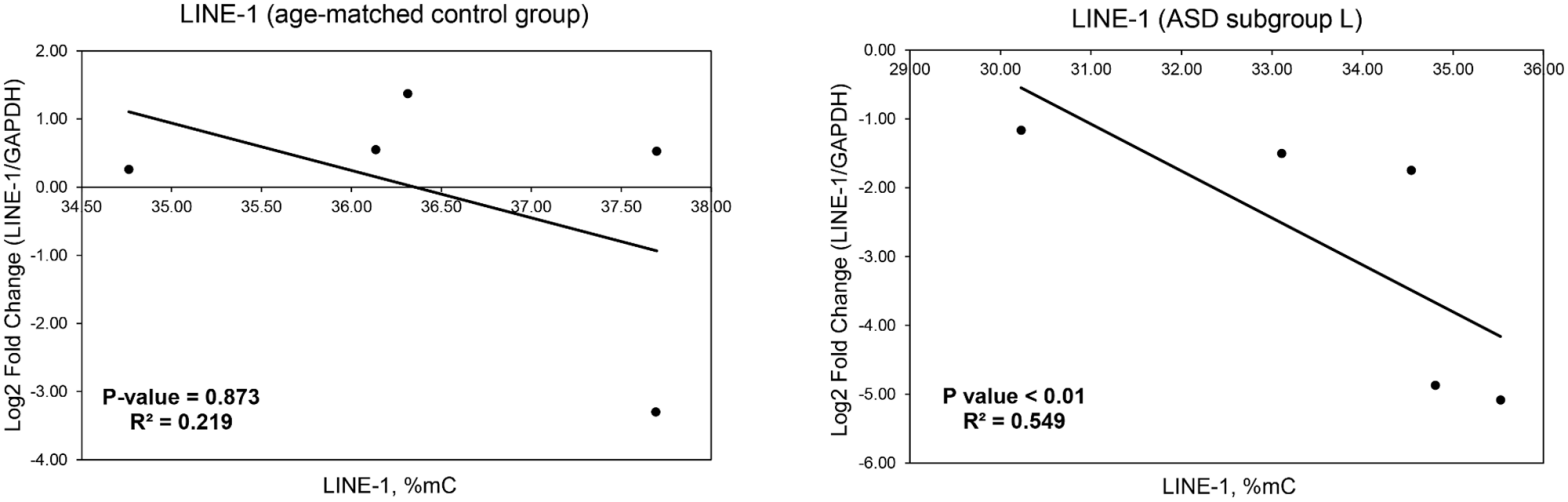
Correlation analysis of LINE-1 methylation and expression in ASD individuals with severe language deficits and sex-/age-matched controls. The X-axis represents the percentage of LINE-1 methylation. The Y-axis represents the log_2_ fold change of LINE-1 expression against *GAPDH*. R^2^ and P-values were calculated via Spearman’s correlation method.

To determine whether the methylation level of LINE-1 is correlated with LINE-1 expression in ASD with severe language deficits, a qRT-PCR analysis of LINE-1 was conducted using 5 ASD and 5 age/sex-matched control LCL samples and an analysis of the correlations between the level of LINE-1 methylation and the expression of LINE-1 was performed. Interestingly, the results showed that LINE-1 expression was modestly reduced in ASD individuals with severe language deficits compared with that in sex/age-matched controls (fold change = 0.148, P-value = 0.049). We subsequently correlated the levels of LINE-1 methylation and expression and found that the expression of LINE-1 in ASD with severe language deficits is inversely correlated with the percentage of overall methylation (%C^m^) of the LINE-1 promoter. No such correlation was observed in the unaffected controls (Fig 5).

**Fig 6:**
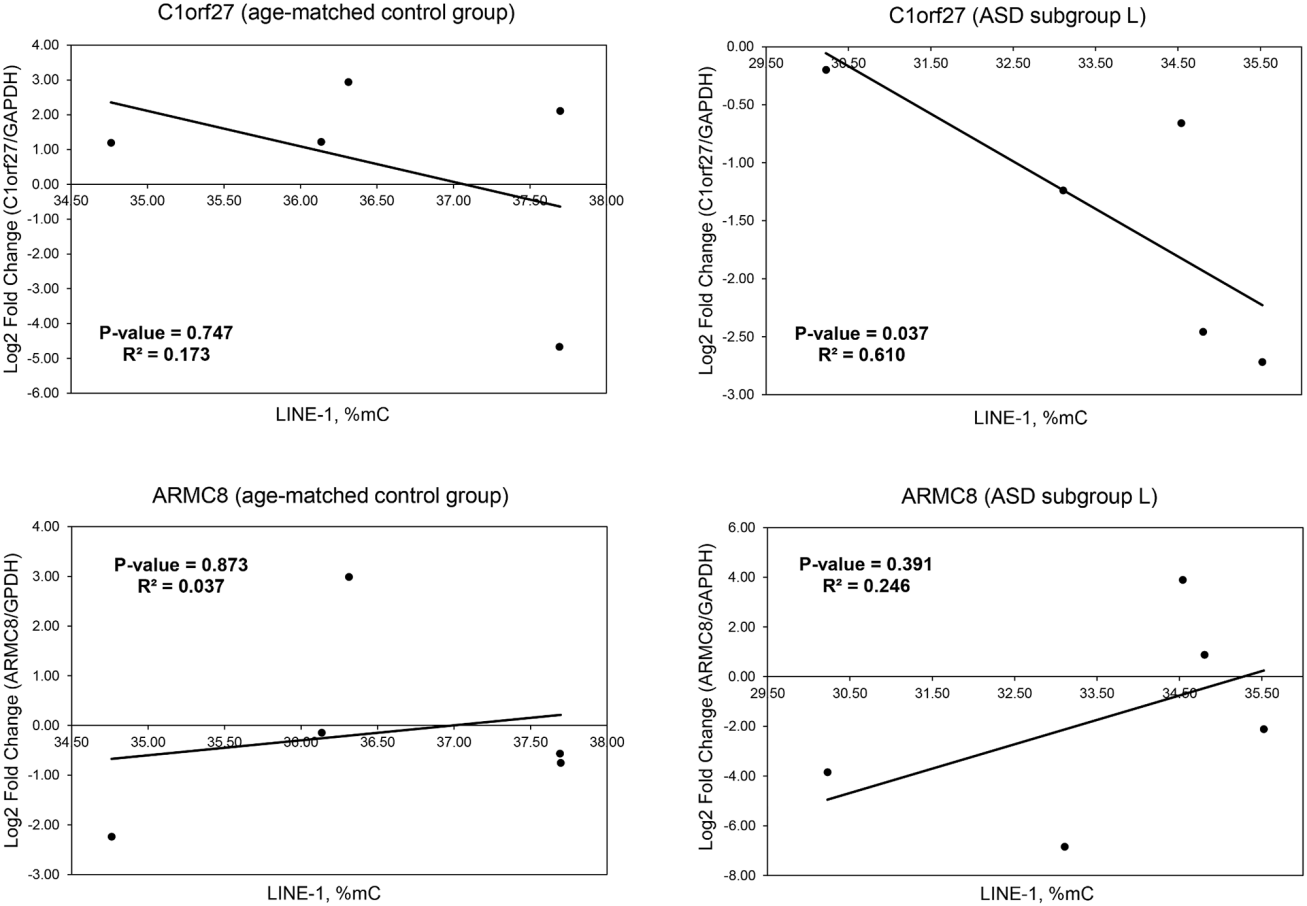
Correlation analysis of LINE-1 methylation and the expression of *C1orf27* and *ARMC8* in ASD individuals with severe language deficits and sex-/age-matched controls. The X-axis represents the percentage of LINE-1 methylation. The Y-axis represents the log_2_ fold change of *C1orf27*/*GAPDH* and *ARMC8*/*GAPDH*. R^2^ and P-values were calculated via Spearman’s correlation method.

Moreover, to determine whether LINE-1 methylation might be associated with differentially expressed genes in ASD with severe language deficits, correlation analysis of global LINE-1 methylation levels and the expression levels of two LINE-1-inserted genes (i.e. *C1orf27* and *ARMC8*) was performed. We selected these two representative genes from the list of downregulated genes in ASD with severe language impairment (subgroup L), which was significantly associated with the LINE-1 insertion types “All insertion” and “Intronic insertion” (P-value < 0.05; Table 3). According to the Human Protein Atlas database (https://www.proteinatlas.org), both genes are highly expressed in the human brain. *C1orf27*, also called *ODR4*, is enriched in the cerebral cortex. In addition to ASD subgroup L, *C1orf27* is also found among the list of overlapping LINE-1-inserted DEGs between the two transcriptomic datasets GSE18123 and GSE42133 (Fig 2 and S4 Table). The function of C1orf27 protein in human is still unclear but is thought to be involved in the trafficking of a diverse set of G-protein coupled receptors, specifically olfactory receptors, allowing for functional expression of the receptors [65, 66]. We are interested in this gene because impairments in olfactory function has been associated with many neurological disorders, including ASD (see review in [67]). *ARMC8*, a gene which is involved in the innate immune system, is highly expressed in the cerebellum, a brain region that has long been implicated in ASD [68]. The role of ARMC8 in the brain is unclear, but a recent study has shown that ARMC8 promotes cell proliferation by upregulating cyclin D1 and MMP7 expression through the canonical Wnt-signaling pathway [69], which is a pathway that have been associated with ASD (see reviews in [70, 71]). Interestingly, similar to the findings regarding LINE-1 expression, the expression of *C1orf27* in ASD individuals with language deficits was inversely correlated with the percentage of overall methylation (%C^m^), but this was not the case in the matched controls (Fig 6). However, the expression of *ARMC8* was not correlated with LINE-1 methylation.

## Discussion

DNA methylation is a major mechanism of epigenetic modification that represses LINE-1 retrotransposons in the human genome. To date, DNA methylation in non-coding regions, including LINE-1 elements, which comprise most of the genome, has yet to be investigated thoroughly in the context of ASD. Aberrations in global DNA methylation of LINE-1 may lead to many alterations in the human genome, including gene expression and LINE-1 retrotransposition [14, 72, 73]. Several studies have demonstrated that LINE-1 is involved in gene expression regulation as LINE-1 retrotransposons contain many transcription binding sites, splicing sites, and polyadenylation signal sequences [74–77]. If a LINE-1 element integrates into or near host genes, its promoter and other signal sequences can drive host gene expression. Moreover, LINE-1 methylation may initiate heterochromatin formation that can spread into host genes [see review in [78]]. However, the association between LINE-1 retrotransposition and altered gene expression in ASD has not been previously studied.

### Enrichment of LINE-1 retrotransposition in differentially expressed genes in ASD

In this study, we first performed an association analysis between dysregulated genes in ASD from five independent transcriptomic studies and LINE-1-inserted genes. We found a significant overrepresentation of genes containing LINE-1 insertion in the lists of differentially expressed genes in whole blood and blood-derived cells (but not LCLs) from individuals with ASD who were subjects of several independent studies (Table 2). Moreover, dysregulated genes showed significant associations with LINE-1 intronic insertion rather than other insertion types. This finding suggests that LINE-1 insertion within an intron of a specific gene may affect gene regulation, which is consistent with a previous study showing that insertion of active LINE-1 into the intron of a host gene could disrupt gene expression *in vitro*. Moreover, unique methylated introns are frequently found in highly expressed genes [14, 79]. We also found that the selected dysregulated genes that were associated with LINE-1 insertion in at least two studies are implicated in autism or comorbid disorders, including intellectual disability, mental retardation, and cognitive impairment (Table 4).

Although as many as 351 DEGs with LINE-1 insertions were found in two or more independent cohorts, it is interesting to note that no DEGs were shared among all five transcriptomic studies (Fig 2). This may be the result of the well-recognized heterogeneity within the ASD population and/or the differences in the sample types used as a model in these studies. Other than the study by Hu et al. [32], the transcriptomic analyses used in our study did not classify ASD individuals into subgroups, or performed a classification using different subtyping strategy. Moreover, there were no clinical phenotypes reported in these studies (specifically ADI-R scores) available for us to perform subgrouping of their respective cohorts. For future studies, classification of ASD individuals into specific subgroups should be performed using a consistent protocol to reduce the heterogeneity of ASD to allow for the overlap analysis between a specific subgroup from multiple studies. For instance, using the subtyping strategy described here (see Table 3), might allow for identification of more DEGs with LINE-1 insertions in individuals with severe language impairment across multiple cohorts.

### Pathways associated with differentially expressed genes containing LINE-1 insertion

In addition to disorders, the IPA also revealed interesting canonical pathways associated with the dysregulated genes containing LINE-1 insertion, such as estrogen receptor signaling and axonal guidance signaling. Regarding estrogen receptor signaling, estrogen is a sex hormone that plays important roles not only in the reproductive organs but also in the brain and neural systems that are known to be implicated in the sex bias of ASD. Estrogen acts through the estrogen receptor, which has two principal forms, estrogen receptor alpha (ERα) and estrogen receptor beta (ERβ) [80]. ERα can affect neurotransmitter systems and is an indicator of neuropsychiatric disorders [81]. ERβ is a major estrogen receptor that is expressed in the brain and acts as an intermediary of estrogen in some processes, such as learning behavior, which is impaired in ASD [59]. Crider et al. studied the regulation of ERβ in the middle frontal gyrus of ASD subjects and suggested that the ERβ signaling network may be impaired in subjects with ASD [58]. Altun et al. further reported that both ER mRNA and protein were reduced in the brain tissues and serum of ASD individuals, suggesting that some pathobiological changes in the brain may be reflected in peripheral tissues [82]. Similarly, *RORA*, which was found to be differentially methylated in LCLs of individuals with ASD, exhibited reduced expression and protein levels in both LCLs and brain tissues of ASD subjects [6], thus supporting the use of peripheral tissues as surrogates for examining biological processes associated with ASD.

Axonal guidance signaling is also highly associated with LINE-1-containing differentially expressed genes. Axon-guidance proteins play an important role in growing axons and neuronal connections during brain development. Alterations of axon-guidance proteins may induce abnormalities in neuronal systems, resulting in neurological disorders [83]. A previous study reported that the expression and protein levels of some axon-guidance receptors, such as roundabout guidance receptor 2 (ROBO2) and plexin A4 (PLXNA4), were significantly decreased in the brains of ASD subjects [54], and loss of function of these receptors affects the production of some neurotransmitters. In addition, interference with axon-guidance receptor activation may result in abnormalities of the corpus callosum, which is involved in problem solving and creativity, and failure of neuronal projections may contribute to neurological disorders [55]. Other canonical pathways that are known to be associated with ASD include ERK/MAPK signaling [53], neurotrophin/TRK signaling [56], synaptic long-term potentiation, CREB signaling in neurons [see review in [57]], PTEN signaling [60], mTOR signaling [61], circadian rhythm signaling [[62] and see review in [63]], and neuroinflammation signaling [64].

### ASD subtype-dependent association of LINE-1 insertion, methylation, and expression

Interestingly, while LINE-1-containing genes were overrepresented among the differentially expressed genes from the four studies involving peripheral blood cells, LINE-1 insertion was not significantly associated with dysregulated genes in LCLs of ASD individuals (from GSE15402). However, the latter study involving LCLs was performed on subphenotypes of ASD to reduce the heterogeneity inherent within the ASD population [32].

Our recent study investigated the methylation of the repetitive element Alu and found that Alu methylation was not significantly different between ASD and sex- and age-matched control groups when all ASD individuals were combined. However, significantly altered Alu methylation patterns were observed in ASD cases sub-grouped based on ADI-R scores compared with those in matched controls, suggesting that classification of ASD individuals into subgroups based on phenotypes may be beneficial and provide insights into the still unknown etiology and underlying mechanisms of ASD [84]. We therefore reanalyzed the transcriptome profile by subtypes of ASD and discovered that only downregulated genes in the severely language-impaired subgroup (subgroup L) were associated with LINE-1 insertion (Table 3). To address our hypothesis that LINE-1 retrotransposition is involved in altered gene expression in ASD, we selected representative LCL samples from ASD subgroup L for determination of LINE-1 methylation levels and patterns that could disrupt gene regulation. Our findings indicated that compared to the matched unaffected control group, ASD subgroup L showed significantly reduced overall LINE-1 methylation (%C^m^), while no differences in overall methylation were found in subgroups M and S or in the combined ASD samples. Moreover, LINE-1 expression was inversely correlated with LINE-1 methylation in subgroup L (Fig 5). We further examined the association of LINE-1 methylation with the expression of two differentially expressed genes in subgroup L. The correlation analysis showed that one of the two selected genes (*C1orf27*) correlated with LINE-1 methylation levels (%C^m^) in LCLs of subgroup L but not in those of sex- and age-matched unaffected controls (Fig 6). These findings suggest that some but not all dysregulated genes containing LINE-1 may be affected by altered LINE-1 methylation, which is dependent on the subphenotype of ASD. Interestingly, a previous study has also demonstrated that the hypomethylation of intragenic LINE-1 repressed gene expression [19]. Although our previous results of LINE-1 methylation levels in ASD subgroup L showed that they were significantly reduced and should result in overexpression of LINE-1 because DNA methylation is responsible for repressing gene expression, our findings demonstrated that the LINE-1 expression level was significantly reduced in ASD subgroup L. The discrepancies between global LINE-1 methylation levels and LINE-1 expression may result from differences in the regulatory mechanisms of LINE-1 expression in ASD individuals and unaffected controls. Our correlation analysis showed that the overall methylation levels of the LINE-1 promoter were correlated with LINE-1 expression only in ASD subgroup L but not in the control group (Fig 5), suggesting that the methylation of these CpG residues may regulate LINE-1 expression only in ASD subgroup L and not in controls. LINE-1 expression in unaffected individuals may be regulated by other transcriptional regulatory mechanisms independent of the methylation of these CpG residues. Alterations of these differential regulatory mechanisms may be involved in the reduction of LINE-1 expression in ASD individuals compared to that in controls regardless of LINE-1 methylation. LINE-1 regulatory mechanisms and the differences in such mechanisms in ASD children and typically developing children deserves further comprehensive investigation. However, Shpyleva et al. (2017) reported that LINE-1 is overexpressed and the binding of MeCP2 to LINE-1 sequences was significantly lower in the ASD cerebellum [30]. Similar to our findings, DNA methylation of the LINE-1 5’UTR, ORF1, and ORF2 tended to be reduced in ASD cerebellum although was not statistically significant, possibly due to the heterogeneity within the ASD population. Interestingly, we found that LINE-1 methylation within 5’UTR showed hypomethylated status only in LCLs of ASD subgroup L but not in all ASD individuals. This finding suggests that classification ASD individuals into subphenotypic groups may provide additional insights into the still unknown etiology of ASD. However, tissue samples used in our study are different from the previous study by Shpyleva et al. (2017). Although our preliminary findings revealed significantly lower LINE-1 methylation in the L subgroup of ASD, further confirmation in a larger cohort of the same clinical subphenotype is needed. In addition, similar studies need to be carried out using brain tissues and/or primary blood cells in order to conclude that LINE-1 methylation is dysregulated and is associated with ASD etiology and/or susceptibility. However, as shown here, the individuals included in any such study should be subphenotyped in order to reduce the heterogeneity for the LINE-1 methylation analyses.

### Limitations and future directions

A possible limitation with respect to detecting significant LINE-1 methylation differences between LCLs from individuals with ASD and that from controls is passage number of LCLs. In this study, we cultured all LCLs in the same batch and employed multiple biological replicates for each group to minimize the effects of passage number on changes in the methylation pattern in LCLs. However, all LCLs used in this study were obtained from the Autism Genetic Resource Exchange (AGRE) repository and the exact passage number of each LCL was not available. Given the possibility that cell passage number might affect the DNA methylation [85], future studies should strive to exclude this confounding factor. Moreover, preferential amplification of unmethylated alleles (i.e. PCR-bias) might occur. In this study, the exact same procedure was therefore used for all DNA samples using the same protocol that has been used in several studies for LINE-1 methylation analysis [21, 22, 51, 52] and has been demonstrated that the results were well correlated with pyrosequencing method [86]. Future studies may include data adjustments using correction methods, such as the method proposed by Moskalev et al. (2011), to reduce the bias [87].

Another limitation of this study is that the global DNA methylation analysis of LINE-1 promoters used in this study does not measure methylation of LINE-1 at specific dysregulated genes that contain LINE-1. Thus, it is possible that signals from significant methylation sites were dampened by noise from other non-significant sites in the genome. LINE-1 methylation within dysregulated genes or at specific genomic locations should be further studied. In addition, we analyzed LINE-1 methylation and expression using specific primers for the promoter of LINE-1 elements. Although these primers were designed to target highly conserved regions of intragenic LINE-1 elements containing a 5’UTR, two open reading frames, and 3’UTR rather than intergenic LINE-1 elements [13, 19] and these techniques have been used in several recent studies in the context of several diseases/disorders [21, 22, 51, 52], we could not demonstrate that the genome of LCLs or the genes of interest (*C1orf27* and *ARMC8*) indeed contain full-length LINE-1 sequences due to the limitations of COBRA and qRT-PCR analyses of LINE-1 in which primers were generally designed to amplify only short and restricted regions. However, it is well-established that full-length LINE-1 sequences are present in human genomes [88, 89], thus likely that LCLs derived from ASD and unaffected individuals also contain full-length LINE-1 sequences from their genomic DNA. Future studies may include whole LINE-1 genomic sequencing to demonstrate that the whole LINE-1 elements are indeed present in the genome and dysregulated in the genes of interest. Moreover, there might be differences in the copy number of LINE-1 elements between individuals within ASD and control groups as reported in a recent study [90]. Our study was therefore focused on conserved LINE-1 regions in the genome, included multiple biological replicates in each group, and used sex- and age-matched controls, all of which can help minimize the effect of such variations. The number of LINE-1 insertions in each individual should be determined in future experiments to assess such variation.

## Conclusion

In conclusion, our findings suggest that LINE-1 insertion is associated with dysregulated genes in whole blood and blood-derived cells from individuals with ASD. These genes are highly associated with ASD as well as comorbid disorders and are involved in ASD-related canonical pathways. Moreover, altered global LINE-1 methylation and expression was found in LCLs from a specific subtype of ASD characterized by severe language impairment and may influence dysregulation of some but not all genes within this subtype. The findings from this pilot study provide additional evidence implicating noncoding LINE-1 retrotransposons and epigenetic modification in gene disruption in ASD. Our study will be useful for designing further experiments with different sample types, such as whole blood, somatic tissue, or brain tissue.

## Supporting information

S1 Table Demographic information of the LCLs used in this study. (DOC)

S2 Table Hypergeometric distribution analysis of the overlap between the list of LINE-1-inserted genes and the list of DEGs from each transcriptomic dataset compared to the list of randomly selected genes equal in number to the respective list of DEGs. P-values were adjusted using Benjamini-Hochberg’s multiple test correction method (FDR < 0.05). The DEG lists with P-values indicating significantly more LINE-1 insertions in the DEG datasets than in the list of randomly selected genes are highlighted in yellow.
(DOCX)

S3 Table Hypergeometric distribution analysis of the overlap between the list of LINE-1-inserted genes and the list of DEGs from the transcriptomic dataset GSE15402 phenotypically subgrouped based on ADI-R scores compared to the list of randomly selected genes equal in number to the respective list of DEGs. P-values were adjusted using Benjamini-Hochberg’s multiple test correction method (FDR < 0.05). The DEG lists with P-values indicating significantly more LINE-1 insertions in the DEG datasets than in the list of randomly selected genes are highlighted in yellow. L = ASD with severe language impairment; M = mild ASD; S = ASD with savant skills.
(DOCX)

S4 Table List of the overlapping genes containing LINE-1 insertion identified in at least two studies. (DOCX)

## Abbreviations

ADI-R: Autism Diagnostic Interview-Revised
ARMC8: Armadillo Repeat Containing 8
ASD: Autism spectrum disorder
AUC: Area under the curve
C1orf27: Chromosome 1 open reading frame 27
COBRA: Combined bisulfite restriction analysis
DNMT: DNA methyltransferases
DSM-5: The Diagnostic and Statistical Manual of Mental Disorders, Fifth Edition
ERα: Estrogen receptor alpha
ERβ: Estrogen receptor beta
EST: Expressed sequence tag
IPA: Ingenuity Pathway Analysis
LCLs: Lymphoblastoid cell lines
LINE-1: Long interspersed element-1
MeCP2: Methyl-CpG binding Protein 2
ORF1: Open reading frame 1
ORF2: Open reading frame 2
PLXNA4: Plexin A4
ROBO2: Roundabout guidance receptor 2
SAM: S-adenosyl-methionine

## References

[1] Christensen DL, Bilder DA, Zahorodny W, Pettygrove S, Durkin MS, Fitzgerald RT, et al Prevalence and Characteristics of Autism Spectrum Disorder Among 4-Year-Old Children in the Autism and Developmental Disabilities Monitoring Network. J Dev Behav Pediatr. 2016;37(1):1–8. 10.1097/DBP.0000000000000235 26651088

[2] Bailey A, Le Couteur A, Gottesman I, Bolton P, Simonoff E, Yuzda E, et al Autism as a strongly genetic disorder: evidence from a British twin study. Psychological medicine. 1995;25(1):63–77. 7792363 10.1017/s0033291700028099

[3] Fombonne E. Epidemiology of autistic disorder and other pervasive developmental disorders. The Journal of clinical psychiatry. 2005;66 Suppl 10:3–8. 16401144

[4] Schaefer GB, Mendelsohn NJ. Genetics evaluation for the etiologic diagnosis of autism spectrum disorders. Genet Med. 2008;10(1):4–12. 10.1097/GIM.0b013e31815efdd7 18197051

[5] Kanherkar RR, Bhatia-Dey N, Csoka AB. Epigenetics across the human lifespan. Front Cell Dev Biol. 2014;2:49 10.3389/fcell.2014.00049 25364756 PMC4207041

[6] Nguyen A, Rauch TA, Pfeifer GP, Hu VW. Global methylation profiling of lymphoblastoid cell lines reveals epigenetic contributions to autism spectrum disorders and a novel autism candidate gene, RORA, whose protein product is reduced in autistic brain. FASEB journal: official publication of the Federation of American Societies for Experimental Biology. 2010;24(8):3036–51. 20375269 10.1096/fj.10-154484 PMC2909294

[7] Wong CC, Meaburn EL, Ronald A, Price TS, Jeffries AR, Schalkwyk LC, et al Methylomic analysis of monozygotic twins discordant for autism spectrum disorder and related behavioural traits. Mol Psychiatry. 2014;19(4):495–503. 10.1038/mp.2013.41 23608919 PMC3906213

[8] Ladd-Acosta C, Hansen KD, Briem E, Fallin MD, Kaufmann WE, Feinberg AP. Common DNA methylation alterations in multiple brain regions in autism. Mol Psychiatry. 2014;19(8):862–71. 10.1038/mp.2013.114 23999529 PMC4184909

[9] Nardone S, Sams DS, Reuveni E, Getselter D, Oron O, Karpuj M, et al DNA methylation analysis of the autistic brain reveals multiple dysregulated biological pathways. Transl Psychiatry. 2014;4:e433 10.1038/tp.2014.70 25180572 PMC4203003

[10] Cordaux R, Batzer MA. The impact of retrotransposons on human genome evolution. Nat Rev Genet. 2009;10(10):691–703. 10.1038/nrg2640 19763152 PMC2884099

[11] Beck CR, Garcia-Perez JL, Badge RM, Moran JV. LINE-1 elements in structural variation and disease. Annual review of genomics and human genetics. 2011;12:187–215. 10.1146/annurev-genom-082509-141802 21801021 PMC4124830

[12] Lander ES, Linton LM, Birren B, Nusbaum C, Zody MC, Baldwin J, et al Initial sequencing and analysis of the human genome. Nature. 2001;409(6822):860–921. 10.1038/35057062 11237011

[13] Penzkofer T, Dandekar T, Zemojtel T. L1Base: from functional annotation to prediction of active LINE-1 elements. Nucleic Acids Res. 2005;33(Database issue):D498–500. 10.1093/nar/gki044 15608246 PMC539998

[14] Han JS, Szak ST, Boeke JD. Transcriptional disruption by the L1 retrotransposon and implications for mammalian transcriptomes. Nature. 2004;429(6989):268–74. 10.1038/nature02536 15152245

[15] Tremblay A, Jasin M, Chartrand P. A double-strand break in a chromosomal LINE element can be repaired by gene conversion with various endogenous LINE elements in mouse cells. Molecular and cellular biology. 2000;20(1):54–60. 10594008 10.1128/mcb.20.1.54-60.2000 PMC85044

[16] Moran JV, DeBerardinis RJ, Kazazian HH Jr. Exon shuffling by L1 retrotransposition. Science. 1999;283(5407):1530–4. 10066175 10.1126/science.283.5407.1530

[17] Newkirk SJ, Lee S, Grandi FC, Gaysinskaya V, Rosser JM, Vanden Berg N, et al Intact piRNA pathway prevents L1 mobilization in male meiosis. Proceedings of the National Academy of Sciences. 2017;114(28):E5635. 10.1073/pnas.1701069114 PMC5514719 28630288

[18] Kitkumthorn N, Mutirangura A. Long interspersed nuclear element-1 hypomethylation in cancer: biology and clinical applications. Clin Epigenetics. 2011;2(2):315–30. 10.1007/s13148-011-0032-8 22704344 PMC3365388

[19] Aporntewan C, Phokaew C, Piriyapongsa J, Ngamphiw C, Ittiwut C, Tongsima S, et al Hypomethylation of intragenic LINE-1 represses transcription in cancer cells through AGO2. PLoS One. 2011;6(3):e17934 10.1371/journal.pone.0017934 21423624 PMC3057998

[20] Kitkumthorn N, Keelawat S, Rattanatanyong P, Mutirangura A. LINE-1 and Alu methylation patterns in lymph node metastases of head and neck cancers. Asian Pacific journal of cancer prevention: APJCP. 2012;13(9):4469–75. 23167363 10.7314/apjcp.2012.13.9.4469

[21] Lertkhachonsuk R, Paiwattananupant K, Tantbirojn P, Rattanatanyong P, Mutirangura A. LINE-1 Methylation Patterns as a Predictor of Postmolar Gestational Trophoblastic Neoplasia. Biomed Res Int. 2015;2015:421747 10.1155/2015/421747 26448937 PMC4584058

[22] Phokaew C, Kowudtitham S, Subbalekha K, Shuangshoti S, Mutirangura A. LINE-1 methylation patterns of different loci in normal and cancerous cells. Nucleic Acids Res. 2008;36(17):5704–12. 10.1093/nar/gkn571 18776216 PMC2553567

[23] Sukapan P, Promnarate P, Avihingsanon Y, Mutirangura A, Hirankarn N. Types of DNA methylation status of the interspersed repetitive sequences for LINE-1, Alu, HERV-E and HERV-K in the neutrophils from systemic lupus erythematosus patients and healthy controls. J Hum Genet. 2014;59(4):178–88. 10.1038/jhg.2013.140 24430577

[24] Macia A, Widmann TJ, Heras SR, Ayllon V, Sanchez L, Benkaddour-Boumzaouad M, et al Engineered LINE-1 retrotransposition in nondividing human neurons. Genome Res. 2017;27(3):335–48. 10.1101/gr.206805.116 27965292 PMC5340962

[25] Muotri AR, Chu VT, Marchetto MC, Deng W, Moran JV, Gage FH. Somatic mosaicism in neuronal precursor cells mediated by L1 retrotransposition. Nature. 2005;435(7044):903–10. 10.1038/nature03663 15959507

[26] Coufal NG, Garcia-Perez JL, Peng GE, Yeo GW, Mu Y, Lovci MT, et al L1 retrotransposition in human neural progenitor cells. Nature. 2009;460(7259):1127–31. 10.1038/nature08248 19657334 PMC2909034

[27] Kuwabara T, Hsieh J, Muotri A, Yeo G, Warashina M, Lie DC, et al Wnt-mediated activation of NeuroD1 and retro-elements during adult neurogenesis. Nat Neurosci. 2009;12(9):1097–105. 10.1038/nn.2360 19701198 PMC2764260

[28] Muotri AR, Marchetto MC, Coufal NG, Oefner R, Yeo G, Nakashima K, et al L1 retrotransposition in neurons is modulated by MeCP2. Nature. 2010;468(7322):443–6. 10.1038/nature09544 21085180 PMC3059197

[29] Mitchell MM, Woods R, Chi LH, Schmidt RJ, Pessah IN, Kostyniak PJ, et al Levels of select PCB and PBDE congeners in human postmortem brain reveal possible environmental involvement in 15q11-q13 duplication autism spectrum disorder. Environ Mol Mutagen. 2012;53(8):589–98. 10.1002/em.21722 22930557 PMC3739306

[30] Shpyleva S, Melnyk S, Pavliv O, Pogribny I, Jill James S. Overexpression of LINE-1 Retrotransposons in Autism Brain. Mol Neurobiol. 2017. 10.1007/s12035-017-0421-x 28220356

[31] Hu VW, Steinberg ME. Novel clustering of items from the Autism Diagnostic Interview-Revised to define phenotypes within autism spectrum disorders. Autism research: official journal of the International Society for Autism Research. 2009;2(2):67–77. 19455643 10.1002/aur.72 PMC2737479

[32] Hu VW, Sarachana T, Kim KS, Nguyen A, Kulkarni S, Steinberg ME, et al Gene expression profiling differentiates autism case-controls and phenotypic variants of autism spectrum disorders: evidence for circadian rhythm dysfunction in severe autism. Autism research: official journal of the International Society for Autism Research. 2009;2(2):78–97. 19418574 10.1002/aur.73 PMC2737477

[33] Sarachana T, Xu M, Wu RC, Hu VW. Sex hormones in autism: androgens and estrogens differentially and reciprocally regulate RORA, a novel candidate gene for autism. PLoS One. 2011;6(2):e17116 10.1371/journal.pone.0017116 21359227 PMC3040206

[34] Sarachana T, Hu VW. Genome-wide identification of transcriptional targets of RORA reveals direct regulation of multiple genes associated with autism spectrum disorder. Mol Autism. 2013;4(1):14 10.1186/2040-2392-4-14 23697635 PMC3665583

[35] Sarachana T, Hu VW. Differential recruitment of coregulators to the RORA promoter adds another layer of complexity to gene (dys) regulation by sex hormones in autism. Mol Autism. 2013;4(1):39 10.1186/2040-2392-4-39 24119295 PMC4016566

[36] Hu VW, Sarachana T, Sherrard RM, Kocher KM. Investigation of sex differences in the expression of RORA and its transcriptional targets in the brain as a potential contributor to the sex bias in autism. Mol Autism. 2015;6:7 10.1186/2040-2392-6-7 26056561 PMC4459681

[37] Hu VW, Addington A, Hyman A. Novel autism subtype-dependent genetic variants are revealed by quantitative trait and subphenotype association analyses of published GWAS data. PLoS One. 2011;6(4):e19067 10.1371/journal.pone.0019067 21556359 PMC3083416

[38] Hu VW. Subphenotype-dependent disease markers for diagnosis and personalized treatment of autism spectrum disorders. Dis Markers. 2012;33(5):277–88. 10.3233/DMA-2012-0916 22960334 PMC3810690

[39] Talebizadeh Z, Arking DE, Hu VW. A Novel Stratification Method in Linkage Studies to Address Inter- and Intra-Family Heterogeneity in Autism. PLoS One. 2013;8(6):e67569 10.1371/journal.pone.0067569 23840741 PMC3694043

[40] Hu VW, Lai Y. Developing a Predictive Gene Classifier for Autism Spectrum Disorders Based upon Differential Gene Expression Profiles of Phenotypic Subgroups. N Am J Med Sci (Boston). 2013;6(3). 10.7156/najms.2013.0603107 PMC3867975 24363828

[41] Edgar R, Domrachev M, Lash AE. Gene Expression Omnibus: NCBI gene expression and hybridization array data repository. Nucleic Acids Res. 2002;30(1):207–10. 11752295 10.1093/nar/30.1.207 PMC99122

[42] Barrett T, Wilhite SE, Ledoux P, Evangelista C, Kim IF, Tomashevsky M, et al NCBI GEO: archive for functional genomics data sets—update. Nucleic Acids Res. 2013;41(Database issue):D991–5. 10.1093/nar/gks1193 23193258 PMC3531084

[43] Alter MD, Kharkar R, Ramsey KE, Craig DW, Melmed RD, Grebe TA, et al Autism and increased paternal age related changes in global levels of gene expression regulation. PLoS One. 2011;6(2):e16715 10.1371/journal.pone.0016715 21379579 PMC3040743

[44] Gregg JP, Lit L, Baron CA, Hertz-Picciotto I, Walker W, Davis RA, et al Gene expression changes in children with autism. Genomics. 2008;91(1):22–9. 10.1016/j.ygeno.2007.09.003 18006270

[45] Kong SW, Collins CD, Shimizu-Motohashi Y, Holm IA, Campbell MG, Lee IH, et al Characteristics and predictive value of blood transcriptome signature in males with autism spectrum disorders. PLoS One. 2012;7(12):e49475 10.1371/journal.pone.0049475 23227143 PMC3515554

[46] Pramparo T, Lombardo MV, Campbell K, Barnes CC, Marinero S, Solso S, et al Cell cycle networks link gene expression dysregulation, mutation, and brain maldevelopment in autistic toddlers. Mol Syst Biol. 2015;11(12):841 10.15252/msb.20156108 26668231 PMC4704485

[47] Levy A, Sela N, Ast G. TranspoGene and microTranspoGene: transposed elements influence on the transcriptome of seven vertebrates and invertebrates. Nucleic Acids Res. 2008;36(Database issue):D47–52. 10.1093/nar/gkm949 17986453 PMC2238949

[48] Saeed AI, Bhagabati NK, Braisted JC, Liang W, Sharov V, Howe EA, et al TM4 microarray software suite. Methods Enzymol. 2006;411:134–93. 10.1016/S0076-6879(06)11009-5 16939790

[49] Wangsri S, Subbalekha K, Kitkumthorn N, Mutirangura A. Patterns and possible roles of LINE-1 methylation changes in smoke-exposed epithelia. PLoS One. 2012;7(9):e45292 10.1371/journal.pone.0045292 23028911 PMC3445447

[50] Sirivanichsuntorn P, Keelawat S, Danuthai K, Mutirangura A, Subbalekha K, Kitkumthorn N. LINE-1 and Alu hypomethylation in mucoepidermoid carcinoma. BMC Clin Pathol. 2013;13:10 10.1186/1472-6890-13-10 23510117 PMC3610265

[51] Chalitchagorn K, Shuangshoti S, Hourpai N, Kongruttanachok N, Tangkijvanich P, Thong-ngam D, et al Distinctive pattern of LINE-1 methylation level in normal tissues and the association with carcinogenesis. Oncogene. 2004;23(54):8841–6. 10.1038/sj.onc.1208137 15480421

[52] Senthong A, Kitkumthorn N, Rattanatanyong P, Khemapech N, Triratanachart S, Mutirangura A. Differences in LINE-1 methylation between endometriotic ovarian cyst and endometriosis-associated ovarian cancer. Int J Gynecol Cancer. 2014;24(1):36–42. 10.1097/IGC.0000000000000021 24304685

[53] Mukaetova-Ladinska EB, Arnold H, Jaros E, Perry R, Perry E. Depletion of MAP2 expression and laminar cytoarchitectonic changes in dorsolateral prefrontal cortex in adult autistic individuals. Neuropathol Appl Neurobiol. 2004;30(6):615–23. 10.1111/j.1365-2990.2004.00574.x 15541002

[54] Suda S, Iwata K, Shimmura C, Kameno Y, Anitha A, Thanseem I, et al Decreased expression of axon-guidance receptors in the anterior cingulate cortex in autism. Mol Autism. 2011;2(1):14 10.1186/2040-2392-2-14 21859478 PMC3177773

[55] Bagri A, Marín O, Plump AS, Mak J, Pleasure SJ, Rubenstein JLR, et al Slit Proteins Prevent Midline Crossing and Determine the Dorsoventral Position of Major Axonal Pathways in the Mammalian Forebrain. Neuron. 2002;33(2):233–48. 11804571 10.1016/s0896-6273(02)00561-5

[56] Correia CT, Coutinho AM, Sequeira AF, Sousa IG, Lourenco Venda L, Almeida JP, et al Increased BDNF levels and NTRK2 gene association suggest a disruption of BDNF/TrkB signaling in autism. Genes Brain Behav. 2010;9(7):841–8. 10.1111/j.1601-183X.2010.00627.x 20662941

[57] Ebert DH, Greenberg ME. Activity-dependent neuronal signalling and autism spectrum disorder. Nature. 2013;493(7432):327–37. 10.1038/nature11860 23325215 PMC3576027

[58] Crider A, Thakkar R, Ahmed AO, Pillai A. Dysregulation of estrogen receptor beta (ERbeta), aromatase (CYP19A1), and ER co-activators in the middle frontal gyrus of autism spectrum disorder subjects. Mol Autism. 2014;5(1):46 10.1186/2040-2392-5-46 25221668 PMC4161836

[59] Bodo C, Rissman EF. New roles for estrogen receptor beta in behavior and neuroendocrinology. Front Neuroendocrinol. 2006;27(2):217–32. 10.1016/j.yfrne.2006.02.004 16603234

[60] Lugo JN, Smith GD, Arbuckle EP, White J, Holley AJ, Floruta CM, et al Deletion of PTEN produces autism-like behavioral deficits and alterations in synaptic proteins. Front Mol Neurosci. 2014;7:27 10.3389/fnmol.2014.00027 24795561 PMC3997048

[61] Yeung KS, Tso WWY, Ip JJK, Mak CCY, Leung GKC, Tsang MHY, et al Identification of mutations in the PI3K-AKT-mTOR signalling pathway in patients with macrocephaly and developmental delay and/or autism. Mol Autism. 2017;8:66 10.1186/s13229-017-0182-4 29296277 PMC5738835

[62] Fadini CC, Lamonica DA, Fett-Conte AC, Osorio E, Zuculo GM, Giacheti CM, et al Influence of sleep disorders on the behavior of individuals with autism spectrum disorder. Front Hum Neurosci. 2015;9:347 10.3389/fnhum.2015.00347 26150777 PMC4471742

[63] Tordjman S, Davlantis KS, Georgieff N, Geoffray MM, Speranza M, Anderson GM, et al Autism as a disorder of biological and behavioral rhythms: toward new therapeutic perspectives. Front Pediatr. 2015;3:1 10.3389/fped.2015.00001 25756039 PMC4337381

[64] El-Ansary A, Al-Ayadhi L. Neuroinflammation in autism spectrum disorders. J Neuroinflammation. 2012;9:265 10.1186/1742-2094-9-265 23231720 PMC3549857

[65] Lehman CW, Lee JD, Komives CF. Ubiquitously expressed GPCR membrane-trafficking orthologs. Genomics. 2005;85(3):386–91. 10.1016/j.ygeno.2004.11.009 15718105

[66] Spehr M, Munger SD. Olfactory receptors: G protein-coupled receptors and beyond. J Neurochem. 2009;109(6):1570–83. 10.1111/j.1471-4159.2009.06085.x 19383089 PMC4455932

[67] Tonacci A, Billeci L, Tartarisco G, Ruta L, Muratori F, Pioggia G, et al Olfaction in autism spectrum disorders: A systematic review. Child Neuropsychol. 2017;23(1):1–25. 10.1080/09297049.2015.1081678 26340690

[68] Bruchhage MMK, Bucci MP, Becker EBE. Cerebellar involvement in autism and ADHD. Handb Clin Neurol. 2018;155:61–72. 10.1016/B978-0-444-64189-2.00004-4 29891077

[69] Xie C, Jiang G, Fan C, Zhang X, Zhang Y, Miao Y, et al ARMC8alpha promotes proliferation and invasion of non-small cell lung cancer cells by activating the canonical Wnt signaling pathway. Tumour Biol. 2014;35(9):8903–11. 10.1007/s13277-014-2162-z 24894675

[70] Kalkman HO. A review of the evidence for the canonical Wnt pathway in autism spectrum disorders. Mol Autism. 2012;3(1):10 10.1186/2040-2392-3-10 23083465 PMC3492093

[71] Kwan V, Unda BK, Singh KK. Wnt signaling networks in autism spectrum disorder and intellectual disability. J Neurodev Disord. 2016;8:45 10.1186/s11689-016-9176-3 27980692 PMC5137220

[72] Lorincz MC, Dickerson DR, Schmitt M, Groudine M. Intragenic DNA methylation alters chromatin structure and elongation efficiency in mammalian cells. Nature structural & molecular biology. 2004;11(11):1068–75. 10.1038/nsmb840 15467727

[73] Wanichnopparat W, Suwanwongse K, Pin-On P, Aporntewan C, Mutirangura A. Genes associated with the cis-regulatory functions of intragenic LINE-1 elements. BMC Genomics. 2013;14:205 10.1186/1471-2164-14-205 23530910 PMC3643820

[74] Belancio VP, Hedges DJ, Deininger P. LINE-1 RNA splicing and influences on mammalian gene expression. Nucleic Acids Res. 2006;34(5):1512–21. 10.1093/nar/gkl027 16554555 PMC1415225

[75] Matlik K, Redik K, Speek M. L1 antisense promoter drives tissue-specific transcription of human genes. J Biomed Biotechnol. 2006;2006(1):71753 10.1155/JBB/2006/71753 16877819 PMC1559930

[76] Athanikar JN, Badge RM, Moran JV. A YY1-binding site is required for accurate human LINE-1 transcription initiation. Nucleic acids research. 2004;32(13):3846–55. 10.1093/nar/gkh698 15272086 PMC506791

[77] Tchenio T, Casella JF, Heidmann T. Members of the SRY family regulate the human LINE retrotransposons. Nucleic Acids Res. 2000;28(2):411–5. 10606637 10.1093/nar/28.2.411 PMC102531

[78] Han JS, Boeke JD. LINE-1 retrotransposons: modulators of quantity and quality of mammalian gene expression? Bioessays. 2005;27(8):775–84. 10.1002/bies.20257 16015595

[79] Ball MP, Li JB, Gao Y, Lee J-H, LeProust EM, Park I-H, et al Targeted and genome-scale strategies reveal gene-body methylation signatures in human cells. Nature Biotechnology. 2009;27:361 10.1038/nbt.1533 19329998 PMC3566772

[80] Mueller SO, Korach KS. Estrogen receptors and endocrine diseases: lessons from estrogen receptor knockout mice. Curr Opin Pharmacol. 2001;1(6):613–9. 11757817 10.1016/s1471-4892(01)00105-9

[81] Herrington DM, Howard TD, Hawkins GA, Reboussin DM, Xu J, Zheng SL, et al Estrogen-receptor polymorphisms and effects of estrogen replacement on high-density lipoprotein cholesterol in women with coronary disease. N Engl J Med. 2002;346(13):967–74. 10.1056/NEJMoa012952 11919305

[82] Altun H, Kurutas EB, Sahin N, Sinir H, Findikli E. Decreased levels of G protein-coupled estrogen receptor in children with autism spectrum disorders. Psychiatry Res. 2017;257:67–71. 10.1016/j.psychres.2017.06.008 28734238

[83] Van Battum EY, Brignani S, Pasterkamp RJ. Axon guidance proteins in neurological disorders. Lancet Neurol. 2015;14(5):532–46. 10.1016/S1474-4422(14)70257-1 25769423

[84] Saeliw T, Tangsuwansri C, Thongkorn S, Chonchaiya W, Suphapeetiporn K, Mutirangura A, et al Integrated genome-wide Alu methylation and transcriptome profiling analyses reveal novel epigenetic regulatory networks associated with autism spectrum disorder. Molecular Autism. 2018;9(1):27. 29686828 10.1186/s13229-018-0213-9 PMC5902935

[85] Grafodatskaya D, Choufani S, Ferreira JC, Butcher DT, Lou Y, Zhao C, et al EBV transformation and cell culturing destabilizes DNA methylation in human lymphoblastoid cell lines. Genomics. 2010;95(2):73–83. 10.1016/j.ygeno.2009.12.001 20005943

[86] Jintaridth P, Mutirangura A. Distinctive patterns of age-dependent hypomethylation in interspersed repetitive sequences. Physiol Genomics. 2010;41(2):194–200. 10.1152/physiolgenomics.00146.2009 20145203

[87] Moskalev EA, Zavgorodnij MG, Majorova SP, Vorobjev IA, Jandaghi P, Bure IV, et al Correction of PCR-bias in quantitative DNA methylation studies by means of cubic polynomial regression. Nucleic Acids Res. 2011;39(11):e77 10.1093/nar/gkr213 21486748 PMC3113592

[88] Boissinot S, Chevret P, Furano AV. L1 (LINE-1) retrotransposon evolution and amplification in recent human history. Mol Biol Evol. 2000;17(6):915–28. 10.1093/oxfordjournals.molbev.a026372 10833198

[89] Scott AF, Schmeckpeper BJ, Abdelrazik M, Comey CT, O’Hara B, Rossiter JP, et al Origin of the human L1 elements: proposed progenitor genes deduced from a consensus DNA sequence. Genomics. 1987;1(2):113–25. 3692483 10.1016/0888-7543(87)90003-6 PMC7135745

[90] Ewing AD, Kazazian HH Jr. High-throughput sequencing reveals extensive variation in human-specific L1 content in individual human genomes. Genome Res. 2010;20(9):1262–70. 10.1101/gr.106419.110 20488934 PMC2928504

