# Supplementary material for: Investigation of epigenetic regulatory networks associated with autism spectrum disorder (ASD) by integrated global LINE-1 methylation and gene expression profiling analyses
Source: PLoS One. 2018 Jul 23;13(7):e0201071. doi: 10.1371/journal.pone.0201071 (PMC6056057; doi:10.1371/journal.pone.0201071)
Supplement: S2 Table — P-values were adjusted using Benjamini-Hochberg’s multiple test correction method (FDR < 0.05). The DEG lists with P-values indicating significantly more LINE-1 insertions in the DEG datasets than in the list of randomly selected genes are highlighted in yellow. (DOCX) [file pone.0201071.s002.docx]

**S2 Table. Hypergeometric distribution analysis of the overlap between the list of LINE-1-inserted genes and the list of DEGs from each transcriptomic dataset compared to the list of randomly selected genes equal in number to the respective list of DEGs.**

| Insertion type | Comparison | GEO datasets | All dysregulated genes | | Up-regulated genes | | Down-regulated genes | |
| --- | --- | --- | --- | --- | --- | --- | --- | --- |
|  |  |  | DEGs (p-value) | Random genes (p-value) | DEGs (p-value) | Random genes (p-value) | DEGs (p-value) | Random genes (p-value) |
| All insertion | ASD vs Control | GSE15402 | 8.06E-01 | 5.83E-02 | 8.72E-01 | 1.63E-01 | 7.32E-01 | 1.26E-01 |
|  |  | GSE18123 | **2.88E-13** | **2.66E-12** | **4.74E-50** | **3.04E-15** | 9.48E-01 | 5.66E-02 |
|  |  | GSE25507 | **5.72E-03** | **2.47E-07** | 8.92E-01 | **1.64E-03** | **5.44E-15** | 5.66E-02 |
|  |  | GSE42133 | **1.27E-04** | **1.14E-02** | **2.46E-06** | **2.12E-02** | **1.67E-04** | 8.07E-02 |
|  |  | GSE6575 | **1.81E-05** | **1.10E-03** | **1.97E-09** | **7.89E-03** | 9.27E-02 | **3.70E-02** |
| Intronic | ASD vs Control | GSE15402 | 7.58E-01 | 6.66E-02 | 8.68E-01 | 1.53E-01 | 6.63E-01 | 1.58E-01 |
|  |  | GSE18123 | **3.66E-14** | **1.33E-12** | **1.83E-50** | **3.27E-15** | 9.05E-01 | **3.87E-02** |
|  |  | GSE25507 | **1.65E-03** | **2.27E-07** | 8.22E-01 | **7.78E-04** | **8.82E-16** | **2.11E-05** |
|  |  | GSE42133 | **6.13E-05** | **1.52E-02** | **3.02E-06** | **1.68E-02** | **5.43E-05** | 1.22E-01 |
|  |  | GSE6575 | **4.88E-06** | **1.81E-05** | **9.00E-10** | **4.51E-03** | 5.59E-02 | **2.99E-02** |
| Exonized | ASD vs Control | GSE15402 | 3.43E-01 | 3.21E-01 | 1.00E+00 | 6.56E-01 | 1.00E+00 | 2.41E-01 |
|  |  | GSE18123 | 6.56E-02 | 5.19E-02 | **3.89E-03** | **1.60E-02** | 2.39E-01 | 7.07E-01 |
|  |  | GSE25507 | 2.61E-01 | 1.92E-01 | 9.78E-01 | 8.60E-01 | **9.65E-03** | **3.52E-02** |
|  |  | GSE42133 | 2.69E-01 | 5.68E-01 | 5.32E-02 | 5.87E-01 | 9.11E-01 | 5.86E-01 |
|  |  | GSE6575 | 6.37E-01 | 4.39E-01 | 1.00E+00 | 6.41E-01 | 2.90E-01 | 3.52E-01 |
| Exonic | ASD vs Control | GSE15402 | 4.79E-01 | 8.78E-01 | 3.29E-01 | 9.66E-01 | 8.39E-01 | 5.70E-01 |
|  |  | GSE18123 | 1.13E-01 | **3.88E-02** | **3.89E-03** | **6.46E-03** | 1.00E+00 | 7.75E-01 |
|  |  | GSE25507 | **3.88E-02** | **4.47E-02** | 9.98E-01 | 4.30E-01 | 5.02E-01 | **1.24E-02** |
|  |  | GSE42133 | 1.36E-01 | 5.22E-01 | 4.00E-01 | 3.50E-01 | 7.78E-02 | 6.50E-01 |
|  |  | GSE6575 | 6.37E-01 | 2.06E-01 | 6.82E-01 | 4.01E-01 | 3.81E-01 | 2.12E-01 |
| Promoter | ASD vs Control | GSE15402 | 1.00E+00 | 1.00E+00 | 1.00E+00 | 1.00E+00 | 6.24E-01 | 1.00E+00 |
|  |  | GSE18123 | 9.48E-01 | 9.25E-01 | 8.58E-01 | 9.77E-01 | 8.36E-01 | 5.84E-01 |
|  |  | GSE25507 | 8.99E-01 | 7.32E-01 | 8.65E-01 | 6.49E-01 | 8.14E-01 | 5.68E-01 |
|  |  | GSE42133 | 4.62E-01 | 4.10E-01 | 6.45E-01 | 8.34E-01 | 2.97E-01 | 1.61E-01 |
|  |  | GSE6575 | 2.46E-01 | 4.43E-01 | 1.15E-01 | 9.42E-01 | 6.89E-01 | 7.68E-02 |
