# Supplementary material for: Investigation of epigenetic regulatory networks associated with autism spectrum disorder (ASD) by integrated global LINE-1 methylation and gene expression profiling analyses
Source: PLoS One. 2018 Jul 23;13(7):e0201071. doi: 10.1371/journal.pone.0201071 (PMC6056057; doi:10.1371/journal.pone.0201071)
Supplement: S3 Table — P-values were adjusted using Benjamini-Hochberg’s multiple test correction method (FDR < 0.05). The DEG lists with P-values indicating significantly more LINE-1 insertions in the DEG datasets than in the list of randomly selected genes are highlighted in yellow. L = ASD with severe language impairment; M = mild ASD; S = ASD with savant skills. (DOCX) [file pone.0201071.s003.docx]

**S3 Table. Hypergeometric distribution analysis of the overlap between the list of LINE-1-inserted genes and the list of DEGs from the transcriptomic dataset GSE15402 phenotypically subgrouped based on ADI-R scores compared to the list of randomly selected genes equal in number to the respective list of DEGs.**

| Insertion type | Comparison | Difference gene expression | | Up-regulated gene expression | | Down-regulated gene expression | |
| --- | --- | --- | --- | --- | --- | --- | --- |
|  |  | Hypergeometric | | Hypergeometric | | Hypergeometric | |
|  |  | DEGs (p-value) | Random genes (p-value) | DEGs (p-value) | Random genes (p-value) | DEGs (p-value) | Random genes (p-value) |
| All insertion | ASD subgroup L vs. C | 0.304 | 0.468 | 0.929 | 0.903 | **1.06E-02** | 0.343 |
|  | ASD subgroup M vs. C | 4.02E-01 | 4.68E-01 | 4.51E-01 | 4.68E-01 | 0.468 | 4.68E-01 |
|  | ASD subgroup S vs. C | 4.96E-01 | 4.68E-01 | 0.468 | 4.68E-01 | 8.70E-01 | 8.51E-01 |
| Intronic | ASD subgroup L vs. C | 0.304 | 0.468 | 0.929 | 0.903 | **1.06E-02** | 4.02E-01 |
|  | ASD subgroup M vs. C | 4.02E-01 | 4.68E-01 | 4.51E-01 | 4.68E-01 | 0.468 | 4.68E-01 |
|  | ASD subgroup S vs. C | 5.04E-01 | 4.68E-01 | 0.468 | 4.68E-01 | 9.03E-01 | 8.51E-01 |
| Exonized | ASD subgroup L vs. C | 4.68E-01 | 4.51E-01 | 3.43E-01 | 4.68E-01 | 9.82E-01 | 4.68E-01 |
|  | ASD subgroup M vs. C | 9.82E-01 | 1.00E+00 | 1.00E+00 | 1.00E+00 | 6.79E-01 | 9.33E-01 |
|  | ASD subgroup S vs. C | 7.97E-01 | 9.39E-01 | 8.51E-01 | 8.51E-01 | 8.51E-01 | 1.00E+00 |
| Exonic | ASD subgroup L vs. C | 4.55E-01 | 4.68E-01 | 4.68E-01 | 7.97E-01 | 4.68E-01 | 4.02E-01 |
|  | ASD subgroup M vs. C | 8.70E-01 | 1.00E+00 | 9.03E-01 | 8.65E-01 | 8.51E-01 | 4.68E-01 |
|  | ASD subgroup S vs. C | 4.68E-01 | 9.39E-01 | 9.04E-01 | 8.51E-01 | 3.43E-01 | 1.00E+00 |
| Promoter | ASD subgroup L vs. C | 4.68E-01 | 6.96E-01 | 6.96E-01 | 8.36E-01 | 5.47E-01 | 7.97E-01 |
|  | ASD subgroup M vs. C | 8.70E-01 | 8.70E-01 | 9.90E-01 | 8.51E-01 | 6.41E-01 | 9.29E-01 |
|  | ASD subgroup S vs. C | 9.29E-01 | 1.00E+00 | 1.00E+00 | 1.00E+00 | 1.00E+00 | 1.00E+00 |
