# Supplementary material for: Investigation of epigenetic regulatory networks associated with autism spectrum disorder (ASD) by integrated global LINE-1 methylation and gene expression profiling analyses
Source: PLoS One. 2018 Jul 23;13(7):e0201071. doi: 10.1371/journal.pone.0201071 (PMC6056057; doi:10.1371/journal.pone.0201071)
Supplement: S4 Table — (DOCX) [file pone.0201071.s004.docx]

**S4 Table. List of the overlapping genes containing LINE-1 insertion identified in at least two studies.**

| **Overlapping of study** | **Number of genes** | **Gene symbol** | | | | | |
| --- | --- | --- | --- | --- | --- | --- | --- |
| GSE18123 GSE25507 GSE42133 | 15 | ING3 | ETF1 | RAB22A | BRPF3 | CREB1 |  |
|  |  | OSBPL8 | CBL | SENP5 | WNK1 | UBE4B |  |
|  |  | NFYA | JARID2 | PTPRE | PAK2 | PTPN11 |  |
| GSE18123 GSE25507 GSE6575 | 4 | MMP16 | ACTN1 | RECK | BACH1 |  |  |
| GSE18123 GSE42133 GSE6575 | 3 | KLHL5 | ZBTB16 | CDC42 |  |  |  |
| GSE25507 GSE42133 GSE6575 | 2 | RUNX3 | PABPC1 |  |  |  |  |
| GSE18123 GSE25507 | 94 | ATP9B | ASXL2 | ARHGAP26 | BAZ2B | PRKCH | NRP1 |
|  |  | TRIM33 | FANCA | PIAS1 | TNFSF8 | ADNP | MAP3K5 |
|  |  | TCL6 | CORO1C | TRIP11 | MKL2 | TLE4 | SLC23A2 |
|  |  | SLC8A1 | NF1 | UBR1 | USP33 | TLR2 | DICER1 |
|  |  | EIF1AX | FAM49A | OGFRL1 | KIF13A | ADAMTSL1 | TBC1D5 |
|  |  | ALCAM | WNT9A | ZCCHC6 | NAB1 | BRWD1 | ACOX1 |
|  |  | SLC6A6 | ORMDL1 | ANKMY1 | NHS | PTEN | LARS |
|  |  | QKI | NOTCH2 | FLCN | KIF3A | ARVCF | ANTXR2 |
|  |  | TMEM33 | CHDH | ZNF493 | LAMP2 | TRIO | PLXNC1 |
|  |  | IDS | ESR1 | MAPK1 | ATXN1 | GPR107 | MTF1 |
|  |  | PCNX | HEXB | USP31 | DDX6 | TIMP2 | PPP2R2C |
|  |  | STK38L | PRKAG2 | SNX27 | KLHL8 | UBR2 | PECAM1 |
|  |  | LYST | UBE2B | PLXDC2 | MSI2 | PARD3 | SPG21 |
|  |  | TLR8 | PLAA | SLC16A6 | RANBP17 | DGCR2 | DYRK1A |
|  |  | MAML3 | C11orf30 | PIGA | RIT1 | HIPK1 |  |
|  |  | USP25 | VDR | NIN | ZNF180 | TOP1 |  |
| GSE18123 GSE42133 | 119 | MYO9B | LANCL2 | OSBPL1A | HGF | RBBP4 | MYCBP2 |
|  |  | EP300 | SVIL | PPP1CB | ARID1A | PDPK1 | NMNAT3 |
|  |  | USP47 | CREBBP | PAN3 | RBMS1 | STX7 | STK4 |
|  |  | C1orf27 | ZNF627 | MAP3K4 | ACTR2 | WDR4 | GSTA3 |
|  |  | MSH3 | ITGA6 | IL1RN | IQGAP2 | KLF12 | ELF2 |
|  |  | SEMA4D | LRIG1 | PTPLB | TUBGCP3 | GALNT7 | MGAT4A |
|  |  | TSC22D1 | ZXDC | DDX59 | DCK | TAF4 | ANKRD12 |
|  |  | KIAA1033 | YES1 | ZNF559 | TRIM23 | VPS13D | GNB5 |
|  |  | WHSC1L1 | WWP1 | AZIN1 | CLN8 | BRD1 | UBQLN1 |
|  |  | CCAR1 | FGD2 | ZNF567 | MYH9 | ATP6V0A4 | RAD51 |
|  |  | EP400 | FN3K | MBP | MBTPS1 | EIF4B | PHF20L1 |
|  |  | PPM1A | NAP1L1 | HOOK1 | MEF2A | PKN2 | PPFIA1 |
|  |  | SON | PTPRC | GNB1 | RCOR3 | SEC23A | CANX |
|  |  | XRN2 | INPP5F | ADPGK | SPTAN1 | SAP130 | SSX2IP |
|  |  | SUV420H1 | ME2 | IFNAR2 | MYNN | USP6 | SMARCA2 |
|  |  | ADRA1A | PPP3CB | ZNF91 | PPIG | SERPINE2 | LGALS8 |
|  |  | AHNAK | SH3BP2 | CASP4 | ZNF540 | RAP1B | ZNF420 |
|  |  | CAMSAP1 | TRIM4 | AAK1 | TLL2 | UBL3 | CTSC |
|  |  | GNAO1 | PLA2G2D | PHKB | FCER1A | HCN2 | ZNF638 |
|  |  | PRPF39 | BIN2 | RANBP9 | BTBD2 | MBNL1 |  |
| GSE18123 GSE6575 | 26 | EIF4G3 | ZFYVE16 | SBNO1 | CD1D | ENAH | ATP2A2 |
|  |  | ST8SIA4 | MIPOL1 | ZNF445 | SPAG16 | SSH1 |  |
|  |  | PRRX1 | PDCD1LG2 | RCBTB1 | XRCC6 | APLP2 |  |
|  |  | HERC1 | TFCP2 | DOCK2 | PAPPA | ETS1 |  |
|  |  | LRIG2 | XPO5 | CTBP2 | ACMSD | KPNA6 |  |
| GSE25507 GSE42133 | 39 | LYZL4 | ANP32A | SYNE1 | EPHA8 | ELOVL4 | PTPN3 |
|  |  | BCL2 | HN1 | SLC7A1 | HRSP12 | EPHA1 | RNF125 |
|  |  | CAMTA1 | BBX | N4BP1 | LGR6 | PGM2 | TUBGCP6 |
|  |  | APPBP2 | SBF1 | FBXO38 | CSN3 | SP1 | WWP2 |
|  |  | BBS1 | FEZ1 | ANXA7 | AGPAT3 | PARP10 |  |
|  |  | CTSB | PLEKHG5 | CACNG6 | NRP2 | LHX6 |  |
|  |  | RNF7 | SP2 | PDK3 | DHX30 | PIP |  |
| GSE25507 GSE6575 | 19 | CD44 | SYNCRIP | IGFBP7 | CHMP4B | GYPB |  |
|  |  | MRAP | SH2D1B | PACS2 | HMGA2 | HNF4A |  |
|  |  | TERF2 | ICA1 | HECW2 | NFIB | CDC5L |  |
|  |  | CDS2 | CSNK2A1 | SEMA7A | SLC15A1 |  |  |
| GSE42133 GSE6575 | 30 | GLTP | GRIK2 | SLC35E1 | WDR1 | FKBP1B | ANK1 |
|  |  | PROSC | POLDIP3 | GORASP2 | YY1 | GTF3C2 | AP3D1 |
|  |  | ASB13 | HDAC3 | CEACAM1 | ZNF585B | ICK | EWSR1 |
|  |  | DSTN | OTOF | INO80 | CDH26 | PRPF4 | ST3GAL3 |
|  |  | CD6 | TDP1 | DDX27 | ACSL5 | CPNE3 | VDAC2 |
